# Novel Pathological Role of hnRNPA1 (Heterogeneous Nuclear Ribonucleoprotein A1) in Vascular Smooth Muscle Cell Function and Neointima Hyperplasia

**DOI:** 10.1161/ATVBAHA.117.310020

**Published:** 2017-09-14

**Authors:** Li Zhang, Qishan Chen, Weiwei An, Feng Yang, Eithne Margaret Maguire, Dan Chen, Cheng Zhang, Guanmei Wen, Mei Yang, Bin Dai, Le Anh Luong, Jianhua Zhu, Qingbo Xu, Qingzhong Xiao

**Affiliations:** From the Department of Cardiology, The First Affiliated Hospital, School of Medicine, Zhejiang University, Hangzhou, China (L.Z., Q.C., F.Y., M.Y., B.D., J.Z., Q. Xu); Centre for Clinical Pharmacology, William Harvey Research Institute, Barts and The London School of Medicine and Dentistry, Queen Mary University of London, United Kingdom (Q.C., W.A., F.Y., E.M.M., D.C., C.Z., G.W., L.A.L., Q. Xiao); Department of Cardiothoracic Surgery, The First Affiliated Hospital of Chongqing Medical University, China (D.C., C.Z.); Key Laboratory of Cardiovascular Diseases, The Second Affiliated Hospital and Key Laboratory of Protein Modification and Degradation, School of Basic Medical Sciences (G.W., Q. Xiao), Guangzhou Medical University, Guangdong, China; and Cardiovascular Division, King’s College London British Heart Foundation Centre, United Kingdom (Q. Xu).

**Keywords:** cell migration, cell proliferation, microRNAs, neointima, RNA-, binding protein, vascular smooth muscle cells

## Abstract

**Objective—:**

hnRNPA1 (heterogeneous nuclear ribonucleoprotein A1) plays a variety of roles in gene expression. However, little is known about the functional involvement of hnRNPA1 in vascular smooth muscle cell (VSMC) function and neointima hyperplasia. In this study, we have attempted to investigate the functional roles of hnRNPA1 in the contexts of VSMC function, injury-induced vessel remodeling, and human atherosclerotic lesions, as well as discern the molecular mechanisms involved.

**Approach and Results—:**

hnRNPA1 expression levels were consistently modulated during VSMC phenotype switching and neointimal lesion formation induced by wire injury. Functional studies showed that VSMC-specific gene expression, proliferation, and migration were regulated by hnRNPA1. Our data show that hnRNPA1 exerts its effects on VSMC functions through modulation of IQGAP1 (IQ motif containing GTPase activating protein 1). Mechanistically, hnRNPA1 regulates IQGAP1 mRNA degradation through 2 mechanisms: upregulating microRNA-124 (miR-124) and binding to AU-rich element of IQGAP1 gene. Further evidence suggests that hnRNPA1 upregulates miR-124 by modulating miR-124 biogenesis and that IQGAP1 is the authentic target gene of miR-124. Importantly, ectopic overexpression of hnRNPA1 greatly reduced VSMC proliferation and inhibited neointima formation in wire-injured carotid arteries. Finally, lower expression levels of hnRNPA1 and miR-124, while higher expression levels of IQGAP1, were observed in human atherosclerotic lesions.

**Conclusions—:**

Our data show that hnRNPA1 is a critical regulator of VSMC function and behavior in the context of neointima hyperplasia, and the hnRNPA1/miR-124/IQGAP1 regulatory axis represents a novel therapeutic target for the prevention of cardiovascular diseases.

## Abstract 2

Supplemental Digital Content is available in the text.

Neointimal hyperplasia is responsible for restenosis after percutaneous coronary angioplasty/stenting. Restenosis results in the recurrence of symptoms associated with a severe cardiovascular event requiring further treatment and consequently higher death rates. Although the use of drug-eluting stents (DES) instead of bare-metal stents and administration of antiplatelet drugs and statins could significantly reduce the rate of major adverse cardiac events and restenosis, angiographic restenosis still occurs in large number of patients after coronary stenting with DES, and no significant difference in terms of the overall mortality rate and recurrent myocardial infarction was observed between DES and bare-metal stents groups.^1–3^ More alarmingly, the most recent long-period follow-up studies (5–6 years) have either reported that there were no significant differences between patients receiving DES and bare-metal stents in the composite outcome of death from any cause and nonfatal spontaneous myocardial infarction^4^ or reported an even higher number for all-cause deaths (particularly with regard to cardiac mortality) in the DES group.^5^ Surprisingly, major adverse cardiac events occurred in almost one-third of the patients receiving DES (sirolimus- or paclitaxel-eluting stents) within a 10-year follow-up.^6^ Therefore, there is a clinical need for developing further means of reducing complications in patients undergoing percutaneous coronary angioplasty. The vascular smooth muscle cell (VSMC) is one of the major cellular components within the normal vessel wall whose principal function is contraction for regulation of vascular tone, controlling blood pressure and blood flow distribution, as well as providing structural integrity. Unlike most mature cells, VSMCs are remarkably plastic and undergo phenotypic modulation or switching in response to environmental cues. In response to the arterial injury-induced release and activation of growth factors,^7^ differentiated/contractile VSMCs within the tunica media of the arterial wall will dedifferentiate into a so-called synthetic state characterized by significant downregulation of a set of genes encoding SMC-restricted contractile proteins with concomitant upregulation of genes involved in the secretion of extracellular matrix (ECM), cell migration, adhesion, and proliferation. It has been widely accepted that such VSMC phenotypic modulation is dysregulated and has been implicated in the pathogenesis of vascular diseases and remodeling, including atherosclerosis, systemic and pulmonary hypertension, aortic aneurysm and dissection, posttransplant vasculopathy, and restenosis after percutaneous coronary intervention.^8^ Therefore, elucidating the mechanisms controlling VSMC phenotype modulation is critical for understanding the pathogenesis and progression of many vascular diseases and ultimately identifying new therapeutic targets to treat these diseases.

Although recent studies have shed light on some of the pathophysiological mechanisms that are involved in VSMC function and behavior, including (de)differentiation, proliferation, migration, adhesion, senescence/apoptosis, contraction, and extracellular matrix synthesis or degradation,^9,10^ all of which are critical regulators during neointima hyperplasia, the mechanisms that coordinate VSMC contractile and synthetic phenotype switching are still poorly understood, and thus, the molecular modulators that link the coordinated responses of VSMCs to arterial injury remain to be fully elucidated. hnRNPA1 (heterogeneous nuclear ribonucleoprotein A1), a nucleocytoplasmic shuttling protein, has emerged as an important modulator of various gene regulatory networks involved in modulating DNA transcription, mRNA splicing, nuclear export, translation and turnover, miRNA biogenesis, telomeres maintenance, and telomerase activity.^11,12^ Importantly, we have recently demonstrated for the first time that hnRNPA1 is a critical regulator for in vitro and in vivo SMC differentiation from stem cells.^13^ However, thus far, there has been no report investigating the functional involvements of hnRNPA1 in VSMC phenotypic switching and neointima hyperplasia. In the present study, we have provided compelling evidence to support that hnRNPA1 is a major regulator of VSMC phenotype switching and, consequently, arterial remodeling.

## Materials and Methods

A detailed description on the Materials and Methods is provided in the online-only Data Supplement.

## Results

### hnRNPA1 Gene Expression Is Associated With VSMC (De)differentiation

**Figure 1.**
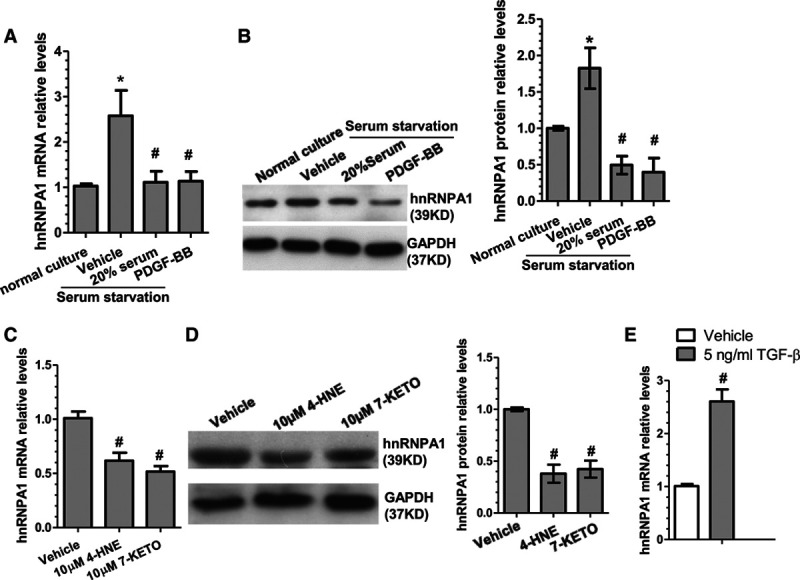
hnRNPA1 (heterogeneous nuclear ribonucleoprotein A1) gene expression is associated with VSMC (vascular smooth muscle cell) phenotypic modulation. **A** and **B**, Quantitative reverse transcription polymerase chain reaction (qRT-PCR) and Western blotting (WB) analysis for hnRNPA1 expression in serum-starved VSMCs restimulated with 0.5% serum (vehicle), 20% serum or 10 ng/mL PDGF-BB (platelet-derived growth factor-BB), respectively, for 48 hours. **C** and **D**, qRT-PCR and WB analysis for hnRNPA1 expression in serum-starved VSMCs restimulated with vehicle, 10 μmol/L 4-hydroxynonenal (4-HNE) or 7-ketocholesteryl (7-Keto), respectively, for 48 hours. **E**, qRT-PCR analysis for hnRNPA1 expression in serum-starved VSMCs restimulated with 5 ng/mL transforming growth factor-β (TGF-β) for 48 hours. The blots in **B** and **D** were subjected to densitometric analysis with Image J software, and the quantitative data of hnRNPA1 protein expression levels (bar graphs, **right**) were included. The data presented here are representative images or mean±SEM of 3 to 5 independent experiments (n=3–5). *P<0.05 (vehicle vs normal culture), #*P*<0.05 (treatment vs vehicle control).

As mentioned earlier, transition of the phenotypic state of VSMCs plays a key role in arterial remodeling and cardiovascular diseases. Therefore, we investigated whether hnRNPA1 played a role in VSMC phenotypic modulation by using well-established in vitro VSMC (de)differentiation models. Data shown in Figure 1 revealed that hnRNPA1 was significantly downregulated during SMC dedifferentiation in response to 10 ng/mL PDGF-BB (platelet-derived growth factor-BB; Figure 1A and 1B), 20% serum (Figure 1A and 1B), and 10 μmol/L ox-LDL (oxidized low-density lipoprotein) components 4-hydroxynonenal and 7-ketocholesteryl (Figure 1C and 1D) treatments, while its expression level was dramatically upregulated when VSMC switch to a contractile differentiated phenotype in response to serum starvation (Figure 1A and 1B) and 5 ng/mL transforming growth factor-β (Figure 1E) stimulation, respectively, suggesting that hnRNPA1 plays a role in VSMC phenotypic modulation.

### hnRNPA1 Mediates VSMC Contractile Gene Expression

Our previous study has reported that hnRNPA1 is a critical regulator for in vitro and in vivo SMC differentiation from stem cells.^13^ To study whether hnRNPA1 also plays a functional role in mature VSMC differentiation and dedifferentiation, hnRNPA1 overexpression and knockdown experiments were conducted in VSMCs using the hnRNPA1-overexpressing plasmid (pCMV5-hnRNPA1) and hnRNPA1 knockdown short hairpin RNAs (shRNAs) generated in our previous study,^13^ respectively. Consistent with our observation in the differentiating stem cells,^13^ the gene expressions of a panel of VSMC contractile proteins (smooth muscle α-actin, calponin, and smooth muscle myosin heavy chain) and VSMC transcriptional factors (serum response factor, myocardin, and myocyte-specific enhancer factor 2C) were significantly upregulated by hnRNPA1 overexpression in VSMCs (Figure IA in the online-only Data Supplement), while its expression levels were dramatically decreased by hnRNPA1 knockdown (Figure IB in the online-only Data Supplement), suggesting that hnRNPA1 also plays a regulatory role in mature VSMC phenotypic modulation.

### VSMC Proliferation and Migration Is Controlled by hnRNPA1

**Figure 2.**
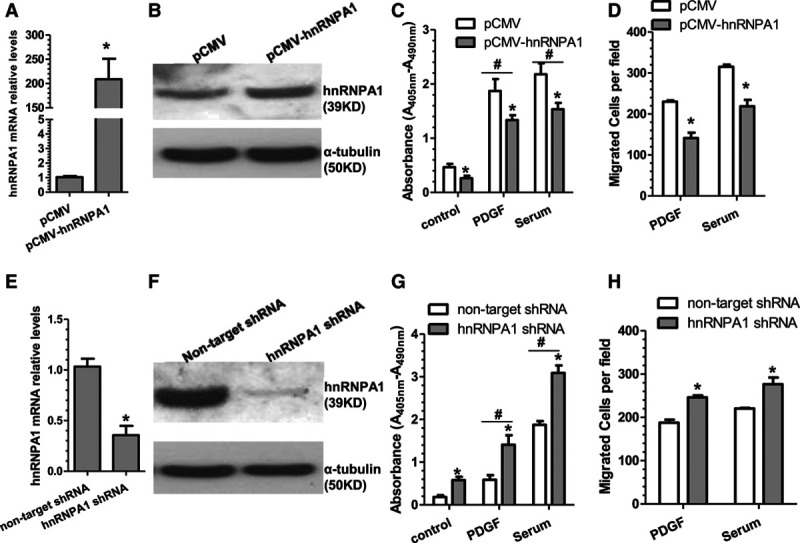
hnRNPA1 (heterogeneous nuclear ribonucleoprotein A1) plays an important role in VSMC (vascular smooth muscle cell) proliferation and migration. **A** and **B**, Quantitative reverse transcription polymerase chain reaction (qRT-PCR) and Western blotting (WB) analysis for hnRNPA1 expression in control and hnRNPA1-overexpressing VSMCs. **C** and **D**, VSMC proliferation and migration were decreased by hnRNPA1 overexpression. **E** and **F**, qRT-PCR and WB analysis for hnRNPA1 expression in control and hnRNPA1 knockdown VSMCs. **G** and **H**, VSMC proliferation and migration were increased by knockdown of hnRNPA1. Note: in the trans-well migration experiments (**D** and **H**), only a few cells were migrated through the insert without any chemoattractant. The data presented here are representative images or mean±SEM of 3 to 5 independent experiments (n=3–5). *P<0.05 (vs control vector [pCMV] or nontarget short hairpin RNA [shRNA]), #*P*<0.05 (stimuli vs control).

To investigate whether hnRNPA1 plays a role in VSMC proliferation and migration, VSMCs were transfected with control or hnRNPA1 overexpression plasmid and subjected to cell proliferation and migration analyses, respectively. Expectedly, the expression levels of hnRNPA1 in VSMCs were significantly upregulated by hnRNPA1 overexpression plasmids (Figure 2A and 2B). Importantly, hnRNPA1 overexpression significantly inhibited VSMC proliferation both at basal level and in response to serum and PDGF-BB stimulation as demonstrated by bromodeoxyuridine incorporation assays (Figure 2C) and cell counting (Figure IIA in the online-only Data Supplement), respectively. A similar effect was observed for VSMC migration (Figure 2D; Figure IIC in the online-only Data Supplement). To further confirm the role of hnRNPA1 in VSMC proliferation, loss-of-function experiments were conducted using hnRNPA1 shRNA in VSMCs, followed by similar treatments and assays. As shown in Figure 2E and 2F, the endogenous levels of hnRNPA1 in VSMCs were significantly downregulated by hnRNPA1 shRNAs. Consequently, VSMC proliferation (Figure 2G; Figure IIB in the online-only Data Supplement), as well as migration (Figure 2H; Figure IID in the online-only Data Supplement) was significantly increased by the knockdown of endogenous hnRNPA1, implying that hnRNPA1 inhibits VSMC growth and migration.

### hnRNPA1 Regulates VSMC Proliferation and Migration Through Inhibition of IQGAP1

**Figure 3.**
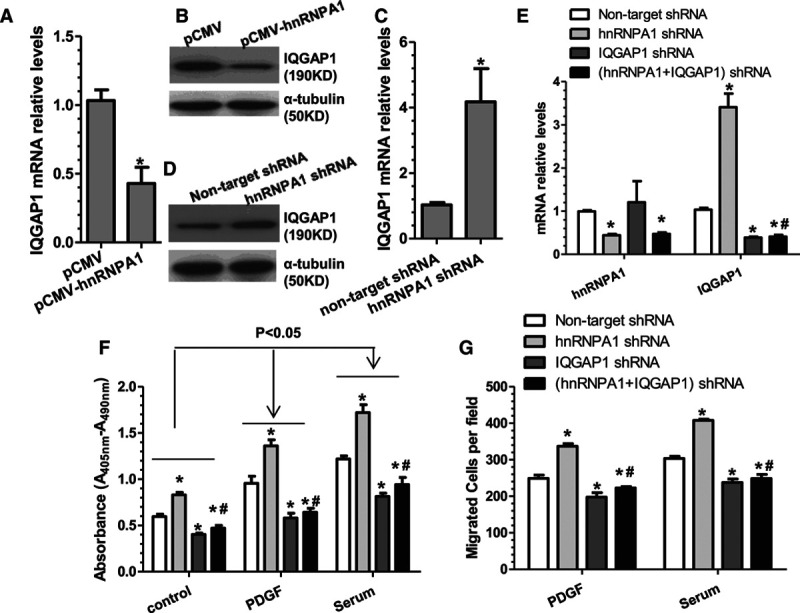
hnRNPA1 (heterogeneous nuclear ribonucleoprotein A1) regulates VSMC (vascular smooth muscle cell) proliferation and migration through inhibition of IQGAP1 (IQ motif containing GTPase activating protein 1). **A**–**D**, Quantitative reverse transcription polymerase chain reaction (qRT-PCR) and Western Blot analyses of IQGAP1 expression in VSMCs with hnRNPA1 overexpression (**A** and **B**) or knockdown (**C** and **D**), respectively. The data presented here are mean±SEM of 3 independent experiments (n=3). *P<0.05 (vs control). **E**, qRT-PCR analysis for hnRNPA1 and IQGAP1 gene expression in VSMCs infected with various short hairpin RNA (shRNA) lentiviruses as indicated. **F** and **G**, IQGAP1 inhibition abolished the promotive effects of hnRNPA1 knockdown on VSMC proliferation (**F**, bromodeoxyuridine incorporation assay) and migration (**G**, trans-well cell migration assay). The data presented here are representative images or mean±SEM of 3 independent experiments (n=3). *P<0.05 (vs nontarget shRNA); #*P*<0.05 (hnRNPA1/IQGAP1 coinhibition vs hnRNPA1 single inhibition, 4th vs 2nd column).

IQGAP1 (IQ motif containing GTPase activating protein 1) has been reported to promote cell proliferation,^14,15^ reduce cell–cell adhesions, increase migration,^16^ and control cellular protrusions, cell shape, and motility by regulating dynamics of actin and microtubule.^17,18^ Particularly, IQGAP1 has been recently reported to play an important role in VSMC migration and neointimal formation.^19^ Therefore, we postulated that hnRNPA1 regulates VSMC proliferation and migration by regulating IQGAP1. Indeed, our data showed that IQGAP1 was significantly downregulated by hnRNPA1 overexpression (Figure 3A and 3B), but upregulated by hnRNPA1 knockdown (Figure 3C and 3D), demonstrating that IQGAP1 is negatively regulated by hnRNPA1 in VSMCs. To further explore the functional implication of IQGAP1 in hnRNPA1-mediated VSMC proliferation and migration, hnRNPA1 and IQGAP1 single- or double-gene knockdown experiments were conducted in VSMCs, followed by VSMC proliferation and migration assays, respectively. Quantitative reverse transcription polymerase chain reaction (qRT-PCR) data showed that both hnRNPA1 and IQGAP1 were successfully downregulated by their respective shRNAs. IQGAP1 gene expression was significantly increased in the hnRNPA1-supressing cells; however, this effect disappeared in VSMCs infected with both the hnRNPA1 and IQGAP1 shRNA lentivirus (Figure 3E). Consequently, proliferation assay data showed that hnRNPA1 or IQGAP1 inhibition alone in the VSMCs significantly increased or decreased VSMC proliferation, respectively. Moreover, increased VSMC proliferation observed in cells undergoing hnRNPA1 inhibition was almost entirely abrogated in cells undergoing simultaneous IQGAP1 suppression (Figure 3F). As expected, similar phenomena were observed with VSMC migration assays (Figure 3G). The above data demonstrate that hnRNPA1 supresses IQGAP1 expression in VSMCs, and such inhibition is required for the decreased VSMC proliferation and migration mediated by hnRNPA1.

### hnRNPA1 Downregulates IQGAP1 Through Upregulation of miR-124

**Figure 4.**
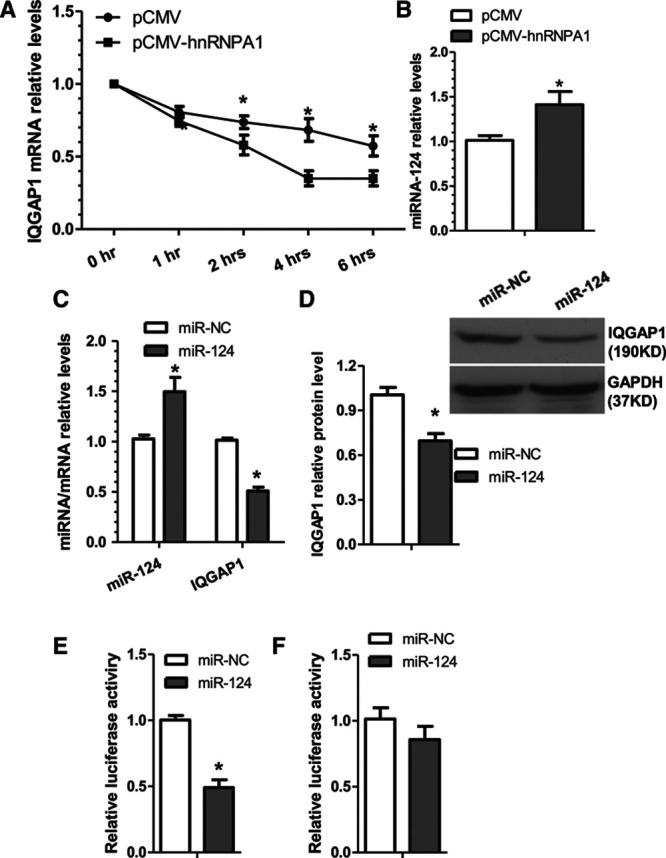
hnRNPA1 (heterogeneous nuclear ribonucleoprotein A1) increases IQGAP1 (IQ motif containing GTPase activating protein 1) mRNA degradation through upregulation of microRNA-124 (miR-124). **A**, hnRNPA1 promotes IQGAP1 mRNA degradation. VSMCs (vascular smooth muscle cells) transfected with control (pCMV) or hnRNPA1 overexpression plasmid (pCMV-hnRNPA1) were treated with an inhibitor of transcription (actinomycin D [ActD], 1 μg/mL) for the indicated times. The IQGAP1 mRNA arbitrary unit at 0 hour for both groups was set as 1.0 and at other time points were calculated accordingly. **B**, miR-124 expression level was significantly upregulated by hnRNPA1. **C** and **D**, IQGAP1 was negatively regulated by miR-124. VSMCs were transfected with miR-124 mimics (miR-124) or microRNA negative controls (miR-NC), respectively. **E** and **F**, IQGAP1 is the target of miR-124, and the miR-124 binding site within IQGAP1 is required for miR-124–mediated IQGAP1 gene repression. miR-124 or miR-NC were cotransfected into VSMCs with wild-type IQGAP1 3′ untranslated region (3′-UTR) reporter (**E**) or the miR-124–binding site mutant (**F**), respectively. Luciferase activity assay were measured at 48 hours post-transfection. The data presented here are representative images or mean±SEM of 3 to 4 independent experiments (n=3–4). *P<0.05 (vs miR-NC).

To elucidate the underlying mechanisms of IQGAP1 downregulation by hnRNPA1 in VSMCs, IQGAP1 mRNA degradation experiments were conducted in VSMCs. Data shown in Figure 4A revealed that in the presence of the new mRNA synthesis inhibitor, actinomycin D, the mRNA stability of IQGAP1 is much lower in VSMCs expressing hnRNPA1 than that of controls cells, suggesting that hnRNPA1 downregulates IQGAP1 through a posttranscriptional mechanism. Moreover, emerging evidence has suggested that microRNAs (miRs) regulate target gene expression at a posttranscriptional level by interacting with the 3′ untranslated regions (3′-UTRs) of the specific mRNAs.^20,21^ Therefore, we wondered whether an miRNA is responsible for IQGAP1 gene repression by hnRNPA1. In this aspect, data from our recent predesigned/customized miR PCR array showed that miR-24 and miR-124 were 2 of the top upregulated miRs during VSMC differentiation into contractile phenotype in response to transforming growth factor-β treatment (Data not shown). Moreover, by closely scrutinizing the 3′-UTR sequence of IQGAP1, we have identified a conserved binding site for both miR-24 and miR-124 within the 3′-UTR of IQGAP1 (Figure IIIA in the online-only Data Supplement). Accordingly, we speculated that hnRNPA1 inhibits IQGAP1 through activation of either miR-24 or miR-124 or both. To this aim, we first assessed whether both miRNAs were modulated by hnRNPA1. qRT-PCR data (Figure 4B; Figure IIIB in the online-only Data Supplement) revealed that overexpression of hnRNPA1 significantly increased both miR-24 and miR-124 expression levels in VSMCs. However, while enforced expression of miR-124 significantly decreased IQGAP1 expression at both mRNA and protein levels (Figure 4C and 4D), no such inhibitory effect was induced by miR-24 overexpression (Figure IIIC in the online-only Data Supplement), indicating that IQGAP1 is negatively regulated by miR-124 but not by miR-24. To determine whether miR-124 can directly regulate IQGAP1, the 3′-UTR of IQGAP1 was cloned into a luciferase reporter using the primers shown in Table I in the online-only Data Supplement. Data from our miRNA reporter assays showed that the activity of luciferase from construct harboring the wild-type IQGAP1 3′-UTR was significantly repressed by miR-124 overexpression (Figure 4E) but not by miR-24 overexpression (Figure IIID in the online-only Data Supplement), providing evidence that IQGAP1 is the target gene of miR-124. As expected, the conserved binding site of miR-124 within the 3′-UTR of IQGAP1 is responsible for miR-124-induced repression of IQGAP1 3′-UTR reporter activity because the expression of miR-124-binding site mutant reporter was not repressed by miRNA-124 overexpression as seen in our luciferase activity assays (Figure 4F). Altogether, the above data demonstrates that IQGAP1 is a true mRNA target of miR-124, and miR-124 is upregulated by hnRNPA1 in VSMCs.

Fluorescent in situ hybridization assay and qRT-PCR analyses revealed that miR-124 is predominantly expressed in VSMCs (≈650 copies per cell; Figure IV in the online-only Data Supplement). To determine whether miR-124 plays a causative role in IQGAP1 gene repression, as well as VSMC proliferation and migration regulated by hnRNPA1, a cotransfection experiment was conducted as indicated in Figure V in the online-only Data Supplement. Gene expression data showed that hnRNPA1 overexpression significantly upregulated miR-124, but this effect was abolished in VSMCs transfected with a miR-124 inhibitor. IQGAP1 expression was significantly inhibited by hnRNPA1 overexpression, but activated by miR-124 inhibition, while its expression level also returned to normal in the cotransfected VSMCs (Figure VA in the online-only Data Supplement). Consequently, VSMC proliferation (Figure VB in the online-only Data Supplement) and migration (Figure VC in the online-only Data Supplement) were significantly inhibited and increased by hnRNPA1 overexpression and miR-124 inhibition, respectively, while such effects were normalized in the cotransfected cells, indicating that miR-124 is at least partially responsible for the effects of hnRNPA1 on IQGAP1 gene repression, as well as VSMC proliferation and migration.

### hnRNPA1 Upregulates miR-124 Through an miRNA Biogenesis Mechanism

**Figure 5.**
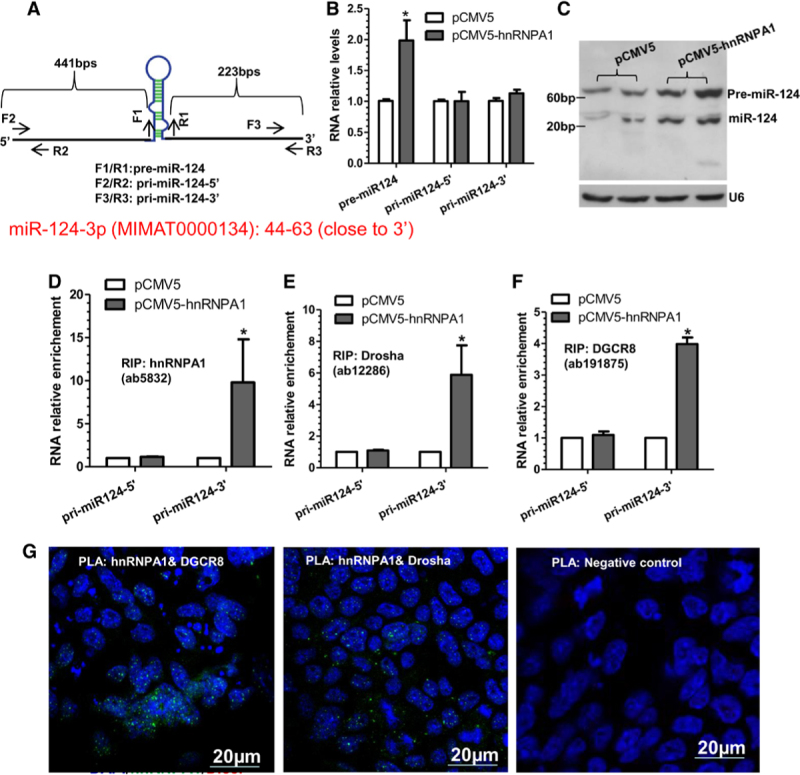
hnRNPA1 (heterogeneous nuclear ribonucleoprotein A1) upregulates microRNA-124 (miR-124) through an miRNA biogenesis mechanism. **A**, Schematic illustration of miR-124 primary transcript and respective primer locations. **B**, Quantitative reverse transcription polymerase chain reaction analysis of miR-124 precursor (pre-miR-124) and primary (pri-miR-124) transcript expression in VSMCs (vascular smooth muscle cells) transfected with control (pCMV) or hnRNPA1 overexpression plasmid (pCMV-hnRNPA1). **C**, Northern Blot analysis of miR-124 and pre-miR-124. **D**–**F**, RNA immunoprecipitation assays were conducted in VSMCs transfected with pCMV or pCMV-hnRNPA1 using antibody against hnRNPA1/HA (**D**), Drosha (**E**), or DGCR8 (DiGeorge syndrome critical region gene 8; **F**), respectively. **G**, Proximity ligation assays using a pair of primary antibodies (or respective IgG controls) as indicated to detect the in situ protein interactions of hnRNPA1 with DGCR8 or Drosha in VSMCs. The data presented here are representative images or mean±SEM of 3 independent experiments (n=3). *P<0.05 (vs pCMV ctrl). RIP indicates RNA immunoprecipitation.

hnRNPA1 is an RNA-binding protein involved in various aspects of RNA processing, and evidence has suggested that hnRNPA1 is required for the biogenesis of some miRNAs.^22,23^ To elucidate the underlying mechanism through which hnRNPA1 upregulates miR-124, we first examined the expression levels of miR-124 primary and precursor transcript in the VSMCs expressing hnRNPA1 and observed that the expression level of the precursor, but not the primary miR-124 transcript, was significantly upregulated by hnRNPA1 overexpression (Figure 5A and 5B). Northern blot analyses also showed that the abundance of the precursor and mature miR-124 was significantly increased by hnRNPA1 overexpression (Figure 5C). Above data imply that hnRNPA1 regulates miR-124 not through a transcriptional mechanism, but via modulating the processing of miR-124 primary transcript to its precursor. Data from RNA immunoprecipitation assays revealed that hnRNPA1, as well as the RNase III Drosha and its coactivator DGCR8 (DiGeorge syndrome critical region gene 8), directly bound to the 3′- but not the 5′-primary miR-124 transcript and that hnRNPA1 overexpression further increased such bindings (Figure 5D through 5F), suggesting that hnRNPA1 participates in miR-124 primary transcript processing into precursor. To further explore the functional role of hnRNPA1 in the regulation of miR-124, proximity ligation assays were conducted in VSMCs with a pair of antibodies (hnRNPA1/Drosha or hnRNPA1/DGCR8). Data from the proximity ligation assays revealed the in situ interactions between hnRNPA1 and Drosha or DGCR8 in VSMCs (Figure 5G). Taken together, above data provide evidence to support a notion that hnRNPA1 binds to the miR-124 primary transcript and recruits both Drosha and DGCR8 into the primary transcript, which works in concert to promote miR-124 processing from primary to precursor transcript.

### hnRNPA1 Downregulates IQGAP1 Through Its Specific Binding Site(s)-AU-Rich Element Within 3′-UTR

AU-rich elements (AREs) have been well-known as one of the most common determinants of RNA stability in mammalian cells, and hnRNPA1 has been reported to specifically bind the ARE within the 3′-UTR of the target mRNA to modulate the target mRNA turnover and translation.^24^ Interestingly, by using the prediction software RegRNA, A Regulatory RNA Motifs and Elements Finder (http://regrna.mbc.nctu.edu.tw/html/prediction.html), or AREsite2 (http://nibiru.tbi.univie.ac.at/AREsite2/welcome), we have identified several AREs within the 3′-UTR of IQGAP1. Importantly, one of such AREs (ATTTA) is adjacent with the reported hnRNPA1 binding site (TAGAG)^11^ and named as hnRNPA1/ARE site (ATTTAGAG; Figure VIA in the online-only Data Supplement). Expectedly, RNA immunoprecipitation assay revealed a direct binding between hnRNPA1 and IQGAP1 transcript (Figure VIB in the online-only Data Supplement). Consequently, enforced expression of hnRNPA1 in VSMCs significantly decreased IQGAP1 3′-UTR luciferase reporter, while such inhibition was clearly impaired when the hnRNPA1/ARE site within 3′-UTR of IQGAP1 was removed (Figure VIC in the online-only Data Supplement), indicating that the hnRNPA1/ARE site is required for hnRNPA1-mediated IQGAP1 gene repression.

### Local Delivery of hnRNPA1 Decreased VSMC Proliferation and Inhibited Neointima Formation in Wire-Injured Carotid Arteries

To study potential involvements of hnRNPA1 in vessel injury–induced neointima formation, a well established arterial remodeling model was used in our study. As expected, we observed that VSMCs underwent phenotypic switch from differentiated to dedifferentiated (proliferative) status after injury as demonstrated by the dramatic decreases in the expression of the SMC-specific contractile genes (eg, *smooth muscle α-actin* and *smooth muscle myosin heavy chain*) as early as day 3 post-injury (Figure VIIA in the online-only Data Supplement) in such process. Moreover, we also found that the expression levels of hnRNPA1 were significantly downregulated during neointimal lesion formation (Figure VIIB in the online-only Data Supplement), suggesting a role for hnRNPA1 in vessel injury–induced neointimal development and progression.

**Figure 6.**
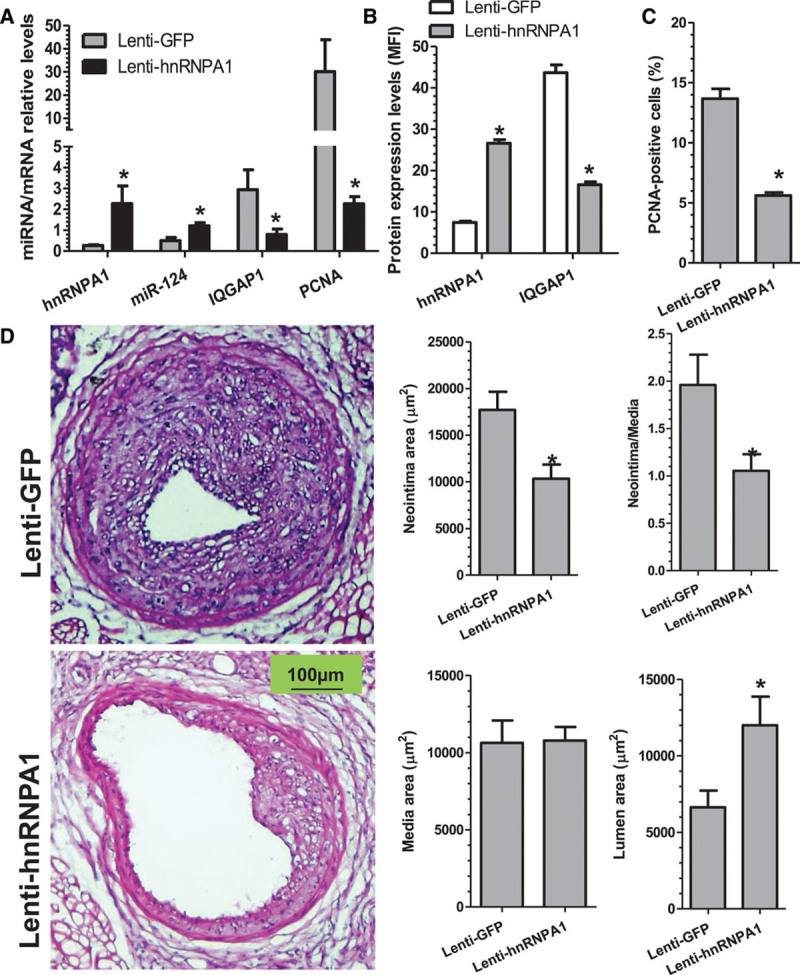
Locally enforced expression of hnRNPA1 (heterogeneous nuclear ribonucleoprotein A1) inhibits neointima formation. **A**–**C**, Vascular overexpression of hnRNPA1 significantly increased the gene expression levels of hnRNPA1 (**A** and **B**) and microRNA-124 (miR-124; **A**), while downregulated the gene expression levels of IQGAP1 (IQ motif containing GTPase activating protein 1; **A** and **B**) and PCNA (proliferating cell nuclear antigen; **A**) and decreased the percentage of PCNA-positive cells (**C**) in the injured vessels. At 7 days (**A**), 14 days (**B** and **C**), or 28 days (**D**) post-treatment, injured segments of carotid arteries were harvested and subjected to qRT-PCR (**A**) and immunofluorescence staining (**B** and **C**) analyses, respectively. The data presented in (**A**) are mean±SEM of 3 independent experiments (carotid arteries from 3 to 5 mice were pooled for each experiment, n=3 experiments). *P<0.05 (Lenti-hnRNPA1 vs Lenti-GFP [green fluorescent protein] mice). Data presented in **B** and **C** are the quantitative data (mean±SEM.) of mean fluorescence intensity (MFI) for the indicated protein (**B**) or the percentage of PCNA-positive cells (**C**) in the injured vessels from 6 mice (n=6 mice for each group). *P<0.05 (vs control mice). **D**, Locally enforced expression of hnRNPA1-inhibited neointima formation in wire-injured carotid arteries. Paraffin sections from both groups (n=15 mice for Lenti-hnRNPA1 and n=14 mice for Lenti-GFP) were prepared and subjected to hematoxylin and eosin (H&E) staining analyses. Representative images (**left**) and morphological characteristics, including media area, neointimal area, neointimal/media (N/M) ratio, and lumen area at 28 days post-injury, were presented here. *P<0.05 (Lenti-hnRNPA1 vs Lenti-GFP).

To investigate the in vivo implication of hnRNPA1 in arterial remodeling, we first generated Lenti-GFP (green fluorescent protein; control) and Lenti-hnRNPA1 lentiviral particles and observed high infection efficiency (>80% of cells were positive for GFP) in primary mouse VSMCs (Figure VIIIA in the online-only Data Supplement). Western blot also confirmed that hnRNPA1 expression was successfully induced in VSMCs by Lenti-hnRNPA1 lentiviral infection (Figure VIIIB in the online-only Data Supplement). Local hnRNPA1 gene delivery was used to determine the potential impact of hnRNPA1 on VSMC growth and neointimal formation in vivo. Ten to 20 μL of DMEM containing 1.0–2.0×10^6^ lentiviral particles (Lenti-hnRNPA1 or Lenti-GFP) was directly infused into the lumen of the injured carotid arteries immediately after injury, followed by a 30-minute incubation period for local VSMC infection. Data from immunofluorescent staining with antibody against GFP showed that majority of VSMCs in the injured vessels were positive for GFP at either 2 (Figure IXA in the online-only Data Supplement) or 4 weeks post-infection (Figure IXB in the online-only Data Supplement), demonstrating that the genes (eg, GFP or hnRNPA1) have been successfully delivered into VSMCs in the injured arteries. Indeed, direct infection of the injured vessels with Lenti-hnRNPA1 significantly increased the expression levels of hnRNPA1 (Figure 6A and 6B; Figure XA in the online-only Data Supplement) and miR-124 (Figure 6A) in the injured arteries compared with the control injured vessels. Moreover, compared with the Lenti-GFP–infected vessels, Lenti-hnRNPA1–infected vessels displayed a much lower level of IQGAP1 at both mRNA (Figure 6A) and protein levels (Figure 6B; Figure XA in the online-only Data Supplement). Furthermore, the gene expression level of PCNA (proliferating cell nuclear antigen) was dramatically decreased in the injured vessels infected with Lenti-hnRNPA1 (Figure 6A). Consistently, lower percentage of PCNA-positive cells was observed within the media and neointima layers of the injured vessels overexpressed with hnRNPA1 (Figure 6C; Figure XB in the online-only Data Supplement), demonstrating that direct infection of the injured vessels with Lenti-hnRNPA1 decreases VSMC proliferation. Consequently, enforced expression of hnRNPA1 in the injured vessels resulted in a nearly 50% decrease in neointima formation after angioplasty (Figure 6D). Data shown in Figure 6D revealed that wire injury of the carotid artery promoted the generation of a thick neointima after 28 days in the mice treated with Lenti-GFP (n=15), which reduced the lumen of the vessel. Such remodeling response was dramatically inhibited by locally enforced expression of hnRNPA1 (Lenti-hnRNPA1, n=14). Specifically, compared with control group, hnRNPA1 overexpression significantly reduced neointimal hyperplasia (neointimal area, 10 500±1500 μm^2^ versus 18 000±1995 μm^2^) and neointima/media ratio (1.09±0.21 versus 1.98±0.32) but dramatically increased the lumen (lumen area, 12 100±2100 μm^2^ versus 6800±1400 μm^2^; Figure 6D) at 28 days post-injury. No significant difference was observed in terms of the medial layer area of carotid arteries between the 2 groups, suggesting a normal arterial contractile state.

### Expression of hnRNPA1-miR-124-IQGAP1 Axis in the Healthy and Diseased Human Vessels

**Figure 7.**
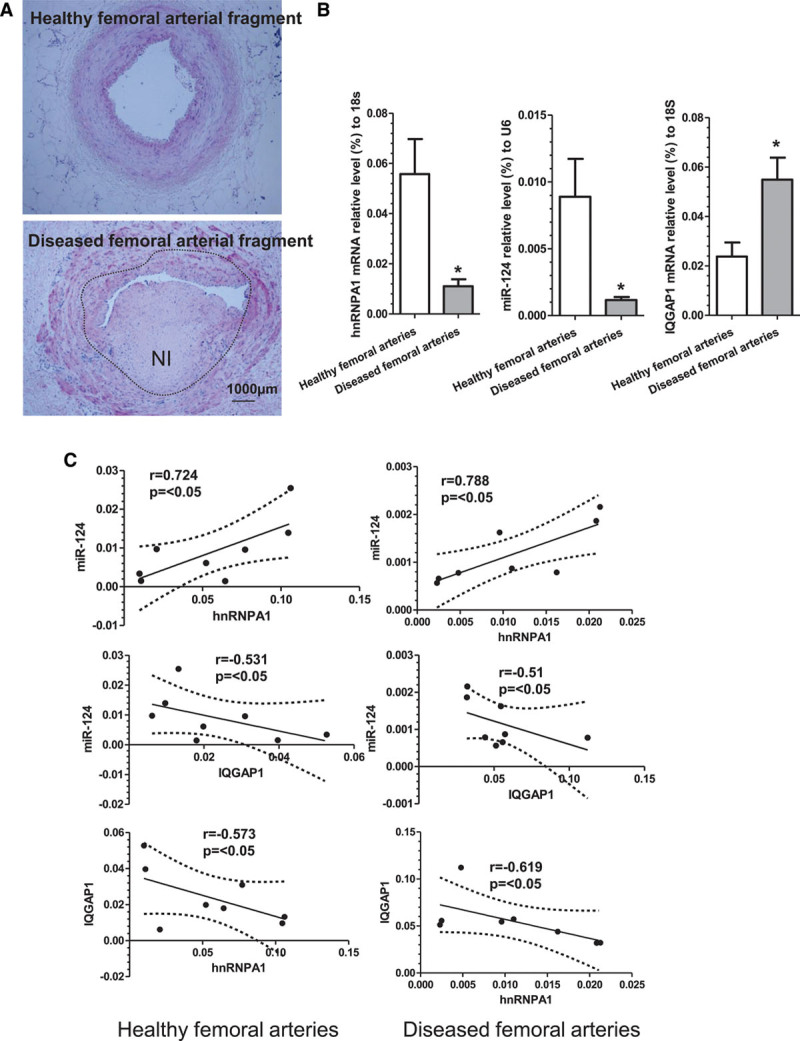
The expression profiles and relationships of hnRNPA1/miR-124/IQGAP1 axis in the human vessels. Eight pairs of femoral arterial specimens (arterial fragments with atherosclerotic lesions (diseased femoral arteries) and their respective neighboring healthy tissues (healthy femoral arteries) from patients with peripheral arterial diseases who underwent leg amputation were collected and subjected to hematoxylin and eosin (H&E) staining (**A**). **B**, Gene expression profiles in normal healthy and diseased femoral arteries. **C**, Pearson correlation coefficient analyses of the gene expression levels of hnRNPA1 (heterogeneous nuclear ribonucleoprotein A1), microRNA-124 (miR-124), and IQGAP1 (IQ motif containing GTPase activating protein 1) in human femoral arterial specimens. The data presented here are representative images or the quantitative data (mean±SEM) of the gene expression for the indicated genes of each group. *P<0.05 (diseased vs adjacent arteries).

To translate our findings from mice to men, 8 pairs of femoral arterial specimens (arterial fragments with atherosclerotic lesions and their respective neighboring healthy tissues) from patients with peripheral arterial diseases that underwent leg amputation were collected at the First Affiliated Hospital of Zhejiang University (China) and subjected to hematoxylin and eosin staining, immunohistochemistry, and qRT-PCR analyses, respectively. All the human vessel specimens were subjected to hematoxylin and eosin staining and examined by 2 independent cardiovascular pathologists. Only the femoral vessel specimens with atherosclerotic lesion and the matched neighboring healthy tissues have been included in the present study (Figure 7A). Data from fluorescent in situ hybridization and immunofluorescent staining show that miR-124 mainly expressed in VSMCs within human femoral arteries (Figure XIA in the online-only Data Supplement). Double immunohistochemistry staining assays also show that VSMCs in human femoral arteries express hnRNPA1 and IQGAP1 (Figure XIB in the online-only Data Supplement). Compared with neighboring healthy tissues, a decreased gene expression level of hnRNPA1 and miR-124, while an increased gene expression level of IQGAP1, was observed in the diseased femoral arteries (Figure 7B). Importantly, a significant inverse relationship between IQGAP1 and hnRNPA1 or miR-124, while a positive correlation between hnRNPA1 and miR-124, was observed in the diseased femoral arterial specimens, as well as neighboring healthy tissues (Figure 7C). The above data provide some critical information about the functional relevance of hnRNPA1 and related downstream targets in human angiographic restenosis.

## Discussion

Adverse arterial remodeling or blood vessel renarrowing after percutaneous coronary intervention remains a significant clinical problem. A greater understanding of the molecular mechanisms underlying abnormal VSMC functions and the pathogenesis of neointimal SMC hyperplasia may provide valuable insight into developing novel therapeutic approaches for postangioplasty restenosis. In the present study, we have advanced our knowledge of the molecular mechanisms governing VSMC proliferation, migration, and neointima formation by documenting an important role of hnRNPA1 in modulating VSMC functions in vitro and in vivo (Figure XII in the online-only Data Supplement). Specifically, various proatherogenic stimuli, including higher concentration of serum, PDGF-BB, 4-hydroxynonenal, and 7-ketocholesteryl, can induce hnRNPA1 downregulation in VSMCs, while VSMC differentiation stimuli, such as serum starvation and transforming growth factor-β, activate hnRNPA1 gene expression. Both VSMC proliferation and migration were modulated by hnRNPA1. Mechanistically, we have demonstrated for the first time that (1) IQGAP1 is the functional downstream target of hnRNPA1 in the context of VSMCs, (2) IQGAP1 repression is required for the inhibitory effects of hnRNPA1 on VSMC proliferation and migration, and (3) hnRNPA1 regulates IQGAP1 through at least 2 regulatory mechanisms: upregulation of miR-124 and via the 3′-UTR of IQGAP1 in which a specific binding site for hnRNPA1 lies. We have also identified IQGAP1 as the mRNA target of miR-124 in VSMCs and demonstrated that hnRNPA1 upregulates miR-124 through an miRNA biogenesis mechanism. Translationally, we have observed that hnRNPA1 expression levels were also downregulated during neointima formation after vessel injury and provided compelling evidence to support that locally enforced expression of hnRNPA1 in injured vessels increases miR-124, represses IQGAP1 gene expression levels, inhibits VSMC proliferation, and prevents postangioplasty restenosis. Finally, we have also provided some preliminary but clear evidence to suggest a role of hnRNPA1/miR-124/IQGAP1 regulatory axis in human angiographic restenosis or atherosclerosis. These findings will significantly increase our understanding of the molecular mechanisms underlying the modulation of VSMC functions in vitro and in vivo and provide a new potential therapeutic target or agent for preventing postangioplasty restenosis.

In human cells, there are over 20 major hnRNPs, named hnRNPs A-U, which are the most abundant nuclear proteins in eukaryotes. hnRNPs are modular proteins consisting of RNA-binding motifs and auxiliary domains characterized by extensive and divergent functions in nucleic acid metabolism. As such, a diverse function of the hnRNPs in gene regulation ranging from nascent transcript packaging to transcriptional regulation, alternative slicing, nucleocytoplasmic transport, and translational regulation of mRNA has been increasingly reported in the literature.^25^ Importantly, by using nuclear proteomics analyses, we observed that the 2 hnRNPs, hnRNPA1^13^ and hnRNPA2B1,^26^ are closely regulated during VSMC differentiation from stem cells. Moreover, it has been widely reported that hnRNPA1 functions as an important modulator in various gene regulatory networks by binding to nascent pre-mRNA,^27^ promoting the annealing of cRNA strands,^28,29^ nuclear export of mature mRNAs,^30^ and mRNA turnover^31^ and translation,^32–34^ and modulating gene alternative splicing by regulating splice site selection,^35–38^ exon skipping or inclusion,^39,40^ and regulating telomere biogenesis/maintenance by directly binding to both single-stranded and structured telomeric DNA repeats.^41,42^ Our recent study has suggested that hnRNPA1 is a critical regulator for in vitro and in vivo SMC differentiation from stem cells through regulation of SMC-specific differentiation gene expression at 2 transcriptional levels: namely at SMC-specific genes and at their transcription factors (serum response factor, myocardin, and myocyte-specific enhancer factor 2C).^13^ We have now provided new evidence to show that hnRNPA1 is also an important regulator for modulating SMC gene expression in mature VSMCs. Both SMC-specific genes (smooth muscle α-actin, h1-calponin, and smooth muscle myosin-11) and their transcription factors (serum response factor, myocardin, and myocyte-specific enhancer factor 2C) were closely modulated by hnRNPA1 as evidenced by their expression levels, which were significantly upregulated by hnRNPA1 overexpression but downregulated by knockdown of hnRNPA1 (Figure I in the online-only Data Supplement). Our previous study^13^ showed that hnRNPA1 regulated SMC gene expression in the differentiating stem cells through a transcriptional mechanism. We speculate that a similar mechanism underpins SMC gene regulation by hnRNPA1 in mature VSMCs. Additionally, our study also provides a new perspective on the role for hnRNPA1 in VSMC biology and vascular pathology. By using gene gain/loss-of-function assays, we demonstrate that hnRNPA1 inhibits both VSMC proliferation and migration, 2 critical cellular events in vascular neointimal lesion formation, which occur in response to higher concentrations of serum or PDGF-BB. Above data support a critical role for hnRNPA1 in VSMC function and behavior. Importantly, by using our well-established model of wire injury–induced neointima formation with local gene delivery into injured vessels, we further demonstrated that locally enforced expression of hnRNPA1 in the injured carotid arteries decreased VSMC growth and prevented neointima SMC hyperplasia after vascular injury, implying that hnRNPA1 is a potential therapeutic agent in postangioplasty restenosis.

One of the major findings in the current study is that IQGAP1 has been identified as the functional downstream target of hnRNPA1 in the context of VSMCs. Our data showed that IQGAP1 promotes VSMC proliferation and migration, and its expression is required for the promotive effects of hnRNPA1 inhibition on VSMC proliferation and migration (Figure 3E through 3G). Importantly, our data showed that IQGAP1 was regulated by hnRNPA1 through 2 distinct mechanisms. On the one hand, hnRNPA1 directly targets and represses IQGAP1 through its specific binding site (hnRNPA1/ARE site: ATTTAGAG, Figure VIA in the online-only Data Supplement) within 3′-UTR of IQGAP1. RNA immunoprecipitation assays show that hnRNPA1 directly binds to IQGAP1 mRNA transcript (Figure VIB in the online-only Data Supplement), and luciferase activity assays with IQGAP1 3′-UTR reporters harboring the wild-type and mutated hnRNPA1/ARE site reveal that such binding site is responsible for hnRNPA1-mediated IQGAP1 gene repression (Figure VIC in the online-only Data Supplement). Therefore, we postulate that hnRNPA1 functions as an AU-element–binding protein, which binds to the hnRNPA1/ARE site within 3′-UTR of IQGAP1, resulting in IQGAP1 mRNA degradation.

On the other hand, our data also show that hnRNPA1 also upregulates miR-124, which in turn promotes IQGAP1 gene repression through its own binding site (GTGCCTTA; Figure IIIA in the online-only Data Supplement) within 3′-UTR of IQGAP1 gene. In this regard, we have provided compelling evidence to support that IQGAP1 is the functional mRNA target of miR-124 in VSMCs. First, close scrutiny of the 3′-UTR sequence of IQGAP1 revealed a conserved binding site for miR-124 within the 3′-UTR of IQGAP1 (Figure IIIA in the online-only Data Supplement). Second, IQGAP1 gene and protein expression levels in VSMCs were negatively regulated by miR-124. Third, overexpression of miR-124 substantially downregulates IQGAP1 3′-UTR activity, but this repression was completely abolished when the miR-124–binging site was mutated. Finally, IQGAP1 gene expression level was inversely associated with hnRNPA1 and miR-124 expression levels in the injured vessels (Figure 6A and 6B), indicating that IQGAP1 is also negatively regulated by hnRNPA1 and miR-124 in vivo.

Another novel finding in the current study that we have demonstrated for the first time is that hnRNPA1 upregulates miR-124 through an miRNA biogenesis mechanism. Increasing evidence has suggested that miR-124 is a tumor suppressor by inhibiting various processes, including cancer cell growth, migration, and invasion.^43–47^ Moreover, one study has indicated that miR-124 controls the proliferative, migratory, and inflammatory phenotype of pulmonary vascular fibroblasts.^48^ More pertinent to our study is that it has recently been reported that miR-124 inhibits the proliferation of pulmonary artery smooth muscle cells by suppressing the transactivation of nuclear factor of activated T cells signaling and targeting multiple genes.^49^ However, the mechanism through which miR-124 is modulated in the context of VSMC functions remains elusive. We found that the mature miR-124 transcript and its precursor, but not the primary transcript of miR-124, was significantly increased by hnRNPA1 overexpression, providing direct evidence to support a notion that hnRNPA1 upregulates miR-124 through an miRNA biogenesis mechanism. It has been widely accepted that RNase III enzyme Drosha and its coactivator DGCR8, a RNA-binding protein, is responsible for processing primary miRs to precursor miRs. Our RNA immunoprecipitation data (Figure 5D through 5F) show that hnRNPA1, as well as DGCR8 and Drosha, specifically bind to 3′ end, but not the 5′ end, of the miR-124 primary transcript, and this binding process is significantly enhanced by hnRNPA1 overexpression. Furthermore, hnRNPA1 was shown to directly interact with Drosha and DGCR8 as demonstrated in our proximity ligation assays (Figure 5F). Altogether, our data show that hnRNPA1 promotes the processing of primary miR-124 to precursor miR-124 through interaction with RNase III enzyme Drosha and its coactivator DGCR8 and recruiting them into miR-124 primary transcript. The concept that hnRNPA1 plays an important role in miR-124 primary transcript processing is consistent with findings from other studies because hnRNPA1 has also been implicated in the regulation of let-7a biogenesis^23^ and is required for processing of miR-18a.^22^

In conclusion, we have shown that hnRNPA1 is a novel regulator of VSMC phenotypic modulation and arterial remodeling, and we have provided compelling evidence to support the notion that modulating the identified downstream target gene (miR-124 and IQGAP1) expression by hnRNPA1 can at least partially explain its effect on VSMC function and injury-induced neointimal formation. However, it is worth mentioning that other downstream genes may also play an important role in hnRNPA1-mediated VSMC phenotype switching and arterial remodeling. One of such genes could be myocardin because data from this study (Figure I in the online-only Data Supplement), as well as our previous study,^13^ show that myocardin is another target gene of hnRNPA1, and Talasila et al^50^ reported that myocardin expression itself is sufficient for modulation of VSMC phenotype and neointimal formation in response to injury. Moreover, several other genes have been identified as the target genes of miR-124 in VSMC context, such as nuclear factor of activated T cell signaling pathway components (nuclear factor of activated T cell c1, calmodulin-binding transcription activator-1, and polypyrimidine tract-binding protein 1),^49^ S100 calcium-binding protein A4,^51^ and Sp1.^52^ Therefore, it would be interesting to further explore whether these target genes can account for the effects of hnRNPA1 on VSMC function and arterial remodeling.

**Highlights**

- hnRNPA1 (heterogeneous nuclear ribonucleoprotein A1) promotes a mature contractile vascular smooth muscle cell phenotype by upregulating vascular smooth muscle cell contractile gene expression and inhibiting vascular smooth muscle cell proliferation and migration.
- hnRNPA1 prevents adverse arterial remodeling and inhibits neointima formation in response to injury.
- hnRNPA1 modulates vascular smooth muscle cell phenotype switching through repressing IQGAP1 (IQ motif containing GTPase activating protein 1) gene expression.
- hnRNPA1 increases IQGAP1 mRNA degradation through 2 mechanisms: increasing microRNA-124 biogenesis and direct binding to the AU-rich element within 3′ untranslated region of IQGAP1.
- We find a decreased gene expression level of hnRNPA1 and microRNA-124 but an increased gene expression level of IQGAP1 in the diseased femoral arteries and observe a significant inverse relationship between IQGAP1 and hnRNPA1 or microRNA-124 and a positive correlation between hnRNPA1 and microRNA-124 in the diseased femoral arterial specimens, as well as neighboring healthy tissues.

## Sources of Funding

This work was supported by British Heart Foundation (FS/09/044/28007, PG/11/40/28891, PG/13/45/30326, PG/15/11/31279, PG/15/86/31723, and PG/16/1/31892 to Q. Xiao) and National Natural Science Foundation of China Grant (91339102, 81270001, 81270180, 81570249, and 91539103). This work forms part of the research portfolio for the National Institute for Health Research Biomedical Research Centre at Barts.

## Disclosures

None.

## Supplementary Material
