# Supplementary material for: Novel Pathological Role of hnRNPA1 (Heterogeneous Nuclear Ribonucleoprotein A1) in Vascular Smooth Muscle Cell Function and Neointima Hyperplasia
Source: Arterioscler Thromb Vasc Biol. 2017 Sep 14;37(11):2182–94. doi: 10.1161/ATVBAHA.117.310020 (PMC5660626; doi:10.1161/ATVBAHA.117.310020)
Supplement: Supplementary file 1 [file atv-37-2182-s001.pdf]

Materials and Methods

Title: A Novel Pathological role of hnRNPA1 in Vascular Smooth Muscle Cell Functions And Neointima Hyperplasia

Li Zhang, et al.

Detailed Methods and Materials

Materials. Antibodies against hnRNPA1 (goat, E17, sc-10030; for Western Blot) and IQGAP1 (mouse, C9, sc-376021) were purchased from Santa Cruz Biotech (USA). Antibodies against hnRNPA1 (mouse, 9H10, ab5832; for immunohistochemistry (IHC) staining and RNA immunoprecipitation (RIP) assays), IQGAP1 (rabbit, EPR5220, ab133490; for IHC staining), Drosha (rabbit, ab12286), DGCR8 (rabbit, EPR18757, ab191875), PCNA (mouse, PC10, ab29) and GAPDH (mouse, 6C5, ab8245) were purchased from Abcam (Cambridge, UK). Antibodies against α-tubulin (mouse, T6074), GFP (mouse, GFP20, G6539), SMA (mouse, 1A4, A5228) and HA (mouse, H3663) were from Sigma (USA). All secondary antibodies were from Dako, Denmark. Other materials used in this study were purchased from Sigma unless specifically indicated.

VSMC culture and treatments

Primary murine VSMCs were isolated from mouse aorta, and routinely maintained in DMEM supplemented with 10% FBS as described in our previous study[1]. As expected, our preliminary data showed that the gene expression levels of VSMC contractile markers were maintained at a comparable level in the cultured VSMC with early passages (up to passage 8), but significantly downregulated in the VSMCs with extended *in vitro* culture (between passage 9 and 12) (Data not shown), therefore VSMCs between passages 3 to 8 were used in the current study. VSMCs were treated with various atherogenic stimuli as described in the previous studies[2-6]. Briefly, for PDGF-BB (Biolegend) and serum stimulation, VSMCs were serum starved for 24~48 hours (0% FBS), followed by an incubation with 20% FBS and 10ng/ml PDGF-BB for 3, 6, 12, 24 and 48 hours, respectively; For TGF-β treatment, VSMCs were serum starved for 24~48 hours, followed by an incubation with 5ng/ml TGF-β (Biolegend) for 24 and 48 hours, respectively; For ox-LDL component treatments, VSMCs were serum starved for 24~48 hours, followed by an incubation with 10µM 4-Hydroxynonenal (4-HNE) and 7-ketocholesterol (7-Keto) for 24 hours.

microRNAs and plasmids transfection

Either microRNA (miR) mimics or miR negative controls (25nM, final concentration) were transfected into VSMCs using TransIT-X2 Transfection Reagent (Geneflow Limited, UK) according to the manufacturer's instructions, and described in our previous study[7]. Briefly, VSMCs ($1.5x10^5$ per well) were seeded into six-well plate 24 hours prior to transfection. Before transfection, cells were washed with 1x PBS once and replenished with 1.75 ml fresh culture medium containing 5% FBS. TransIT-X2 reagent was warmed to room temperature (RT). 250µl of serum free DMEM was added to a sterile Eppendorf tube. Five µl of miR-124 (or miR-24) mimics or control scramble miR mimics (miR-NC) (10µM in stock) was added and mixed using a pipette. 7.5µl of TransIT-X2 reagent was added to the mix and mixed gently. This was left to incubate at RT for 15-30 mins to allow the complexes to form. The TransIT-X2: miRNA complexes were added drop-wise in circular motions to

ensure all the cells being covered by the mixture. The 6-well plate was rocked back-and-forth and side-to-side to evenly distribute the TransIT-X2: miRNA complexes onto the cultured cells. The transfected cells were cultured for 16-24hrs prior to medium change or serum starvation. All miR mimics and miR NC were purchased from Sigma.

For plasmid transfection, control (pCMV5-HA) and hnRNPA1 overexpression (pCMV5-HA-hnRNPA1) plasmids generated in our previous study[8] were transfected into VSMCs using TurboFect Transfection Reagent (Thermo Fisher Scientific Inc.) according to the manufacturer's instructions.

For hnRNPA1 over-expression plasmid and miR-124 inhibitor co-transfection, VSMCs were co-transfected with pCMV5-hnRNPA1 over-expression plasmid (1.0µg per 10^6 VSMCs), a miR-124 inhibitor (25nM, final concentration), and/or respective controls (pCMV5 or miRNA inhibitor negative control) as indicated in the figures, using TransIT-X2 Transfection Reagent (Geneflow Limited, UK) according to the manufacturer's instructions.

IQGAP1 3'UTR clone and their mutants

Reporter vector harboring sequences of the murine IQGAP1 was created using cDNA from VSMCs. The 3'-flanking untranslated region (3'UTR, 5152nt~6333nt) of murine IQGAP1 gene (NM_016721) was amplified by PCR with primers shown in **Table S1** and cloned into the Sac I and Mlu I sites of the pmiR-reporter-basic vector (Ambion, Applied Biosystems), designated as pmiR-Luc-IQGAP1-WT.

miR-124 binding site or hnRNPA1 binding site/AU rich element (A1/ARE) mutations were introduced into pmiR-Luc-IQGAP1-WT by using QuikChange™ site-directed mutagenesis kit (Agilent Technologies) and their respective mutant primers (**Table S1**), according to the manufacturer's instructions. The resultant vectors were designated as pmiR-Luc-IQGAP1-BS$^{mut\text{-}miR124}$ and pmiR-Luc-IQGAP1-BS$^{mut\text{-}A1/ARE}$, respectively.

All vectors and mutants were verified by DNA sequencing.

Transient transfection and luciferase activity assays

Luciferase assays for IQGAP1 3'UTR reporters and its mutants were conducted as described in our previous studies[9-11]. Briefly, VSMCs were cultured in 24–well plates overnight and co-transfected with individual reporter gene (pmiR-Luc-IQGAP1-WT, pmiR-Luc-IQGAP1-BS$^{mut\text{-}miR124}$, or pmiR-Luc-IQGAP1-BS$^{mut\text{-}A1/ARE}$, 0.15 µg/well) and miR-124 mimics (or miR-24 mimics, 25nM per well), or pCMV-hnRNPA1 (0.20 µg/well), or their respective controls, using TransIT-X2 Transfection Reagent (Geneflow Limited, UK), according to the manufacturer's instructions. pmiR-Luc-β-gal (0.20µg/well) was included in all transfection assays as internal control. Luciferase and β-galactosidase activities were detected 48 hours after transfection using a standard protocol. Relative luciferase unit (RLU) was defined as the ratio of Luciferase versus β-galactosidase activity with that of the control (set as 1.0).

shRNA lentiviral particle generation, and infection

While hnRNPA1 shRNA lentiviral particles were generated from our previous study[8], IQGAP1 shRNA lebtiviral particles were produced using MISSION shRNA hnRNPA1 plasmids DNA (SHCLNG-NM_016721, Sigma) according to protocol provided. shRNA lentiviral infection and hnRNPA1 (or IQGAP1) stable knockdown

VSMC generation were performed as described in our previous studies with some modifications[8, 12, 13]. Briefly, VSMCs were plated 24 hours prior to infection in 6 well-plates at 37°C. One transducing Unit per cell (or 2-3x10^5/well) of shRNA or control virus were added with 10µg/ml hexadimethrine bromide (H9268; Sigma). Viral constructs were incubated 24 hours with the cells before the media was replaced with complete media containing 4µg/ml puromycin (P9620, Sigma). For selection of transductants, fresh media containing puromycin was added at 2-3 day intervals for 10 days. Stably infected cells were split and frozen for future experiments.

Generation of hnRNPA1 over-expression pseudoviral particles and infection of VSMCs

The coding region of murine hnRNPA1 gene (NM_001039129) was amplified by PCR with primers shown in **Table S1** and cloned into the EvoR I and Not I sites of the pCDH-CMV-MCS-EF1-Puro vector (CD510B-1, System Biosciences), designated as pCDH-hnRNPA1. Similarly, full length of GFP was amplified by PCR with primers shown in **Table S1** from pEGFP-C1 (632470, Clontech) and cloned into the EvoR I and BamH I sites of the pCDH-CMV-MCS-EF1-Puro vector and designated as pCDH-GFP. All vectors were verified by DNA sequencing.

Control (pCDH-GFP) or hnRNPA1 (pCDH-hnRNPA1) pseudoviral particles were generated using pCDH-GFP or pCDH-hnRNPA1 and packaging plasmids, respectively, according to protocol provided with some modifications. Briefly, 293T cells were co-transfected with the pCDH-GFP or pCDH-hnRNPA1 and the packaging plasmids, psPAX2 (Plasmid #12260, Addgene) and pMD.2 (pCMV-VSV-G, Plasmid #12259, Addgene) using TurboFect Transfection Reagent (Thermo Fisher Scientific Inc.), according to the manufacturer's instructions. The supernatant containing the pseudoviral particles was harvested 48h later, filtered, aliquoted and stored at –80°C. For *in vivo* study, pseudoviral particles was purified and concentrated (100x) using Lenti-X Concentrator (631232, Clontech), according to the manufacturer's instructions. The titers of pseudoviral particles were determined using the Global UltraRapid Lentiviral Titer Kit (LV961A-1, System Biosciences) according to the protocol provided, and the multiplicity of infection (MOI) was calculated using a Standard Curve. Finally, the number of pseudoviral particles in our viral suspension (infection units/ml or IFU/ml) can be calculated with the following equation: (MOI of the sample) X (The number of cells in the well upon infection) X 1000/(µl of viral suspension added to the well for infection).

pCDH-GFP or pCDH-hnRNPA1 lentiviral infection was performed as described above. Briefly, VSMCs were plated 24 hours prior to infection in 6 well-plates at 37°C. One IFU per cell (or 1~2x10^5/well) of pCDH-GFP or pCDH-hnRNPA1 virus were added with 10µg/ml hexadimethrine bromide (H9268; Sigma). Viral constructs were incubated 24 hours with the cells before the media was replaced with complete media. 48~72 hours post-infection, cells were inspected using fluorescence microscopy or were harvested to detect the expression level of hnRNPA1. The infection efficiency of pCDH-GFP or pCDH-hnRNPA1 in mouse VSMCs was shown in **Figure S8**.

VSMC proliferation assays

Cell counting

VSMCs were plated (3.5x10^4 per well) and cultured in 24 well plates pre-coated with 0.04% gelatin and supplemented with complete culture medium containing DMEM, 10% FBS and 1% Penicillin/streptomycin-glutamine. The plates were placed in

humified incubators at 37˚C and 5% CO_2. After culturing for 24hrs, the cells were transfected with pCMV or pCMV-hnRNPA1 plasmid as indicated in the figures. After 12-16 hrs of transfection, the cells were starved by culturing them in the absence of FBS (DMEM supplemented with 1% Penicillin/streptomycin-glutamine) for further 24 hrs. After starvation process, the cells were treated with 20%FBS or PDGF-BB (10ng/ml) for 48hrs before trypsinizing and manually counting the cells under hematocytometer.
For hnRNPA1 shRNA knockdown experiments, VSMCs ($3.5 x 10^4$ per well) infected with non-target or hnRNPA1 shRNA lentiviral particles were cultured in 24-well plates as described above overnight, followed by serum starvation for 24 hrs and various treatment before cell counting.

BrdU incorporation assay

VSMCs were transfected with plasmids or infected with lentiviral particles as described above, and were re-cultured ($0.75 x 10^4$ per well) in 96 well plates overnight, followed by serum starvation for 24 hours. Starved VSMCs were re-stimulated with 20% FBS or 10ng/ml PDGF-BB, respectively, for 48 hours. Cell proliferations were evaluated using 5-Bromo-2'-deoxy-uridine (BrdU) Labeling and Detection Kit II (Roche) according to the manufacturer's instructions. Briefly, cells were incubated with BrdU at a final concentration of 10µM for 12h before measurement. After fixation, cellular DNA was digested by nuclease and labeled with a peroxidase-conjugated BrdU antibody, followed by incubation with the peroxidase substrate. The absorbance of the samples was measured by a microplate reader at 405nm (OD405) with reference measurement at 490nm (OD490). Absorbance (A_{405nm}-A_{490nm}) values representing cell proliferation ability were compared between treatments.

VSMC migration assays

Wound healing (Scratch model)

Scratch wound healing assays were carried out using a previously described method[14]. In brief, VSMCs were cultured on 12-well plates overnight, and transfected with pCMV or pCMV-hnRNPA1 plasmids as described earlier. After 12-16 hrs of transfection, the confluent cells were starved by culturing them in the serum-free DMEM for further 24 hrs. After starvation process, the cells were treated with hydroxyurea (2mM) to inhibit cell proliferation for 2hrs before subjecting them to 20%FBS or PDGF-BB (10ng/ml) treatment. The cells were scratched in criss-cross manner and rinsed with PBS or DMEM three times to remove cell debris, followed by cultured in DMEM supplemented with 20% FBS or PDGF-BB (10ng/ml) in the presence of 2mM hydroxyurea. The observations were made and photomicrographic images were taken at 0hr and 24 hrs, respectively. ImageJ software was used to measure the denuded cell surface of each wound (criss-cross) by two experienced investigators blinded to the treatments, and the percentages of cell closures (migrated area) were calculated as the denuded area difference between hour 0 (A0) and hour 24 (A24) over the denuded area at hour 0, then times 100 [or (A0-A24)/A0*100].
For hnRNPA1 shRNA knockdown experiments, VSMCs infected with non-target or hnRNPA1 shRNA lentiviral particles were cultured in 24-well plates until confluent. Cells were serum-starved for 24 hrs, and subjected to similar treatment and assay as described above.

Trans-well migration assay

VSMCs infected with shRNA lentiviral particles, or transfected with plasmids as described earlier were cultured in FBS-free DMEM for 24 hours, and harvested by trypsinization. An aliquot (250,000 cells/200µl) of the cells in serum-free DMEM was

dispensed into the trans-well inserts (8µm pore size, Greiner Bio-One Ltd, UK. Item number: 662638) pre-coated with 0.5% gelatin (Sigma, G1393), and DMEM with 20% FBS or 30ng/ml PDGF-BB was placed in the lower chamber. The trans-well plates were incubated at 37°C in a 5% CO2 incubator for 12~18 hours. Non-migrated cells in the top insert were carefully removed by cotton swab, and the migrated cells in the bottom side were stained with Crystal Violet dye. Images were captured at five fixed locations (right, bottom, left, up and centre), and migrated cells were counted by two experienced investigators blinded to the treatments.

Immunoblotting

Cells were harvested and lysed in lysis buffer (50mM Tris-Cl pH 7.5, 150mM NaCl, 1 mM EDTA pH 8.0) supplemented with protease inhibitors and 0.5% Triton and sonicated to obtain whole cell lysate. 40 µg of protein was separated by SDS-PAGE with 4%~20% Tris-Glycine gel (Invitrogen, Carlsbad, CA, USA) and subjected to standard Western blot analysis. In some experiments, the blots were subjected to densitometric analysis with Image J software. Relative protein expression level was defined as the ratio of target protein expression level to α-tubulin expression level with that of the control sample set as 1.0.

Real time quantitative PCR (RT-qPCR) for mRNA and microRNAs

Real-time quantitative PCR (RT-qPCR) was performed as previously described[9-11]. Briefly, total RNA containing small RNAs (microRNAs) was extracted from cells using TRI reagent (Sigma) according to the manufacturer's instructions, and subjected to DNase I (Sigma) digestion to remove potential DNA contamination. Reverse transcription for long RNA was performed using an Improm-II™ RT kit (Promega, Madison, WI, USA) with RNase inhibitor (Promega), and Random primers (Promega). The NCode™ VILO™ miRNA cDNA Synthesis Kit (Invitrogen, A11193-051) was used to synthesise poly (A) tails of all the miRNAs followed by cDNA synthesis from the tailed population in a single reaction. The resultant cDNA was diluted to a working concentration of 5ng/µl and stored at -20ºC. NCode™ EXPRESS SYBR® GreenER™ qPCR SuperMix Universal was used in miRNA RT-qPCR. Relative mRNA or microRNA expression level was defined as the ratio of target gene expression level or microRNA expression level to 18S or U6 snRNA expression level, respectively, with that of the control sample set as 1.0. Primers were designed using Primer Express software (Applied Biosystems) and the sequence for each primer was listed in **supplementary Table S1**.

Detection of miR-124 copy number in cells

The miR-124 cope numbers in various cells were detected by RT-qPCR. Briefly, 10^8 copies of synthetic miR-124 input was used to generate cDNA, and a ten-fold serial dilution ranging from 10^8 copies to 10 copies cDNA was amplified by RT-qPCR to generate miR-124 standard curve (CT values versus miR-124 copies). The concentration of total RNAs including small RNAs isolated from cultured cells (10^6 cells) were quantified and 1µg of total RNAs was converted to cDNA (the cDNA was diluted to 200µl to make a final concentration of 5ng/µl). 10ng (2 µl, 1/100 of total cDNA) of cDNA were used in RT-qPCR analyses to obtain the CT values, and the miR-124 copy number (A) was obtained using miR-124 standard curve. Accordingly, miR-124 copy numbers (n) per cell were calculated with a formula: $n=A \times 100 \times DF \times 10^{-6}$, where DF=total RNA amount in µg per 10^6 cells.

Northern blot analyses

The miR-124 precursor and mature transcripts were detected using a highly sensitive miRNA Northern blot assay kit (NB-101, Signosis, Inc.), according to the protocol provided with the kit. Briefly, 10µl (5µg) of total RNAs was separated by 15% pre-run urea-polyacrylamide gel and transferred onto the *Membrane*. The *Membrane* was baked at 80°C for four hours to immobilize the RNAs. The *Membrane* was then put into 50ml Corning disposable tube, with 4ml pre-warmed *NB-hybridization buffer* (NB-HB). The tube was rotated at 42°C for half an hour. The *Membrane* was incubated with 4ml fresh NB-HB containing 10µl biotin-labelled miR-124 (HP-0049, Signosis, Inc.) or U6 (HP-1001, Signosis, Inc.) probe at 42°C overnight. After hybridization, the *Membrane* was rinsed with a wash buffer, and incubated with 4ml fresh NB-HB containing 8µl *amplifier* at 42°C for two hours. After extensive wash, the *Membrane* was transferred to a clean container and washed with 1x NB detection buffer, followed by incubation with 15ml *NB Blocking Buffer* for 30 minutes. Subsequently, the *Membrane* was incubated with 15ml *Streptavidin-Blocking Buffer solution* for further 45 minutes at room temperature (RT) with moderate shaking. After washing with 15mL of 1X *Detection Wash Buffer* three times (3 x 10 minutes), the *Membrane* was incubated with 1.8mL of *Substrate Solution* for 5 minutes. After exposing the *Membrane* with a Hyperfilm for 2~10 minutes, the signal was detected using regular X-ray film developer.

Fluorescent *in situ* hybridization (FISH)

miR-124 expression in VSMCs and tissues was detected by FISH using a miRCURY LNA™ miR-124 Detection Probe and TSA Plus Fluorescence Kit according to the manufacturers' instructions. For FISH detection of miR-124 in VSMCs within human arterial tissues, Dual-stain by FISH (for miR-124) and immunohistochemistry (IHC) (for SMαA) in a double fluorescence assay was conducted. Briefly, paraffin embedded slides (sections) were deparaffinized and rehydrated using Xylene (3 x 5 min) and Ethanol (99.9%, 5 min; 96%, 5 min and 70%, 5 min). Deparaffinized sections were washed in PBS for 5 min, and incubated with 3% H_2O_2 for 15min, followed by incubation with Proteinase-K (20ug/L) at 37°C for 10 min. After washing twice in PBS, slides were transferred into a hybridization container, and incubated with 50 µL hybridization mix containing 40 nM (3.2 µl) double-fluorescein LNA™ miR-124 (EXIQON, 619867-330; mmu/hsa-miR-124-3p probe sequence: GGCATTCACCGCGTGCCTTA) or scramble-miRNA (EXIQON, 699004-330; scramble-miRNA probe sequence: GTGTAACACGTCTATACGCCCA) probe at 60°C for 60 min. After which, the slides were washed in SSC buffer at 60°C (5xSSC, 5 min, once; 1xSSC, 5min, twice; 0.2xSSC, 5 min, twice), and finally in 0.2xSSC buffer at RT for 5 min. Slides were placed in a humidifying chamber and blocked with blocking solution (5%BSA) for 15 min. Subsequently, slides were incubated with HRP-conjugated anti-fluoresecin antibody (1:100 in 1% BSA blocking solution, Abcam, ab6656) and Cy3-conjugated anti-SMαA antibody (1:200 in 1% BSA blocking solution, Sigma, C6198) at 4°C overnight. After washing in PBST (3 min x 3), slides were incubated with TSA™ Fluorescein System (Perkin Elmer, NEL741001KT) at RT for 10 min, and then mounted with SlowFade® Gold antifade reagent with DAPI (Life Technologies, S36938). For FISH detection of miR-124 in cultured VSMCs, cells were fixed in 4% paraformaldehyde/PBS and permeabilized with 0.05% triton-X100/PBS for 15min at RT, respectively. After washing in PBS for 5 min, cells were incubated with 3% H_2O_2 for 15min, washed in PBS (3 min x 3), and treated with Proteinase-K (4ug/L) for a few seconds. The subsequent steps were similar to that of human arterial tissues as described above.

RNA immunoprecipitation (RIP) assays.

The procedure for RNA-IP assays was similar to that described in our previous study[10]. Briefly, VSMCs transfected with pCMV-HA or pCMV-HA-hnRNPA1 were treated with 1% (v/v) formaldehyde at room temperature for 10 min and then quenched with glycine at room temperature. Cells were harvested, lysed and sonicated in the lysis buffer containing RNase inhibitor. The sheared samples were diluted into 1 ml immunoprecipitation buffer, and immunoprecipitations were conducted with 5µg antibodies against hnRNPA1 (ab5832, Abcam) (or HA, H3663, Sigma), Drosha (ab12286, Abcam), DGCR8 (ab191875, Abcam), or equal amount of mouse (or rabbit) IgG, together with protein-G Dynabeads® saturated with single-strand salmon sperm DNA. The immunoprecipitates were digested with DNAse and eluted from the beads using 100 µl elution buffer, and immunoprecipitaed RNA was extracted, purified, and then used to amplify target RNA sequences by RT-qPCR using specific primers (**Table S1**). RNA enrichment with specific antibody was calculated using percent input method with that of the IgG control set as 1.0. PCR amplification of the 5'-end of miR-124 primary transcript or 18s was included as additional control for specific RNA enrichment. The respective target RNAs were almost undetectable or with much higher CT value in normal IgG control samples. The data was obtained from three to four independent experiments.

Proximity Ligation Assays (PLA).

Proximity Ligation Assays (PLA) were conducted using Duolink® reagents (which included a pair of PLA probes-one PLUS and one MINUS, the Detection Reagents, Wash Buffers and Mounting Medium) from Sigma, following the protocol provided in the reagent kits. Briefly, VSMCs were fixed with 4% paraformaldehyde (PFA) and permeabilized with 0.1%Triton X-100 in PBS for 15-30 minutes at room temperature (RT). After washed with PBS twice, cells were blocked with 10% donkey serum for 30 minutes at RT, and incubated with antibodies against hnRNPA1 (mouse, 1:100)/Drosha (rabbit, 1:100), hnRNPA1 (mouse, 1:100)/DGCR8 (rabbit, 1:100), or mouse IgG/rabbit IgG, at 4ºC overnight. Cells were then washed and subsequently incubated with the Duolink® *In Situ* PLA® Probe Anti-Mouse PLUS Affinity purified Donkey anti-Mouse IgG (H+L)/Duolink® In Situ PLA® Probe Anti-Rabbit MINUS Affinity purified Donkey anti-Rabbit IgG (H+L) (1:5 in 10% donkey serum in PBS) in a pre-heated humidity chamber for 1 hr at 37°C. After washing with PBS twice, cells were incubated with Ligation-Ligase solution in a pre-heated humidity chamber for 30 minutes at 37°C, followed by incubation with Amplification-Polymerase solution in a pre-heated humidity chamber for 100 minutes at 37°C. After which, the cells were washed three time in PBS and stained with 4,6-diamidino-2-phenylindole (DAPI) (1ug/ml) for 5 minutes at RT. After mounting, cells were examined using a laser scanning confocal microscope (Zeiss LSM 510 Mark 4) and Zen 2009 image software.

Animal experiments, anaesthesia and euthanasia

All experiments were conducted according to the Animals (Scientific Procedures) Act of 1986 (United Kingdom). In addition, the principles governing the care and treatment of animals, as stated in the Guide for the Care and Use of Laboratory Animals published by the National Academy of Sciences (eighth ed., 2011), were followed at all times during this study. All mice were euthanized by placing them under deep anaesthesia with 100% O_2/5% isoflurane, followed by decapitation.

Mouse carotid artery denudation injury and lentiviral particle infusion

Male JAX™ C57BL/6J Mice (Charles River, UK) were anesthetized and the surgical procedure was similar to that described previously[1, 15-17]. Briefly, the left common carotid artery was dissected and injured by passing a curved flexible wire (0.38-mm diameter, Reference Part Number: C-SF-15-20, Cook Medical European Shared Services, Ireland) three to five times. After the vascular injury, the injured carotid arteries were randomly received control lentivirus (Lenti-Ctrl), lenti-GFP or lenti-hnRNPA1 treatments. The procedures for local gene delivery were similar to that described in the previous studies[18-24] with some modifications. In brief, immediately after injury, 10~20µl of DMEM containing 1.0~2.0 x 10^6 lentiviral particles (Lenti-Ctrl, Lenti-hnRNPA1 or Lenti-GFP) was directly infused into the lumen of the injured carotid arteries, followed by a 30-minute incubation for local VSMC infection. Additional carotid arteries harvested at seven days after injury (3~5 carotid arteries from each group were pooled for each independent experiment, triplicate experiments were conducted) were lysed with TRI reagent (Sigma), and total RNAs including small RNAs were extracted for examining the gene expression levels in the injured vessels using RT-qPCR analysis. Our preliminary experiments revealed that infusion of 1.0~2.0 x 10^6 hnRNPA1 lentiviral particles (lenti-hnRNPA1) generally resulted in 5~10 times higher expression levels compared with control mice (received lenti-GFP infusion), or normalized hnRNPA1 expression levels in injured arteries to the levels similar to normal uninjured vessels (data not shown). All animal experiments were performed according to protocols approved by the Institutional Committee for Use and Care of Laboratory Animals.

Morphometric analysis, quantification of lesion formation, and tissue immunofluorescence staining

The carotid arteries (~5.0mm from injury site) were harvested at 1, 2 or 4 weeks post-operation, respectively. The specimens were fixed in 4% formaldehyde for H&E staining. Sections (8µm) were collected at 200µm intervals (10 sections per segment/interval), mounted on slides, and numbered. Five digitised sections with same identification number from five segments/intervals (~0.5mm, 1.5mm, 2.5mm, 3.5mm and 4.5mm from injury site) of each animal (e.g. I-1/2, III-1/2, V-1/2 represent the 1st and 2nd section of the 1st , 3rd and 5th segment/interval, respectively) were stained with H&E for morphometric analysis. The procedure used for lesion quantification was similar to that described in our previous studies[1, 7, 16, 17]. Briefly, EEL (external elastic membrane), IEL (internal elastic membrane), lumen, media, and neointimal areas were automatically measured on H&E stained cross sectional carotid artery segments using a computerized image analysis system (µm^2, Axiovision software) by two experienced investigators blinded to the treatments. Five sections were analyzed per vessel sample and averaged.

For immunofluorescence staining, three digitised paraffin sections with same identification number from same segment/interval (e.g. I-3, III-3, V-3) of each animal were deparaffined with xylene and rehydrated with ethanol, and then incubated with 10mM sodium citrate at 100˚C for 10 minutes to retrieve antigens, followed by incubation with 1% bovine serum albumin (BSA) for 30 minutes. Thereafter, the sections were incubated with indicated primary antibodies (GFP, hnRNPA1, IGGAP1 or PCNA, all were 1:100 dilutions) or respective IgG controls diluted in blocking buffer in a cold room (4°C) overnight. The tissue sections were then washed and subsequently incubated with an appropriate fluorescence-conjugated 2nd antibody

(1:400 dilution), followed by nuclei staining with 4,6-diamidino-2-phenylindole (DAPI) (1ug/ml). After mounting, the slides were examined using a laser scanning confocal microscope (Zeiss LSM 510 Mark 4) and Zen 2009 image software. The mean fluorescence intensity (MFI) for red fluorescence signal from each section was measured with Image J pro software. Three sections were analyzed per vessel sample and averaged.

Human health and diseased arteries collection and immunohistochemistry analysis

Human femoral arterial specimens were obtained from patients with peripheral arterial diseases undergoing leg amputation at the First Affiliated Hospital of Zhejiang University (China) between July 2014 and June 2017. All patients gave their written, informed consent. All procedures had local ethical approval (2014/294). All studies were approved by the Research Ethics Committees of the First Affiliated Hospital of Zhejiang University and all experiments were conducted according to the principles expressed in the Declaration of Helsinki. To obtain neighbouring healthy arterial tissues, the femoral arteries with maximum length (from three to five centimetres) were harvested. All the human arterial specimens were numbered and divided into five portions and fixed with 4% formaldehyde for H&E staining and/or immunohistochemistry (IHC) assays. All the human vessel specimens were subjected to H&E staining and examined by two independent cardiovascular pathologists. Only the femoral arterial tissues that contained both normal healthy (displaying a normal anatomical structure of blood vessel but absence of atherosclerotic lesion) and diseased (>50% arterial lumen was occluded by atherosclerotic lesions) arterial tissues were included in the present study.

Total RNAs including small RNAs were isolated from the identified and paired paraffin-embedded (FFPE) tissues using a miRCURY™ RNA Isolation Kits (FFPE) (EXIQON, 300115), and subjected to a standard RT-qPCR analysis for gene expression.

Sections of formaldehyde-fixed paraffin-embedded blocks of femoral arteries were subjected to double immunostaining for the VSMC marker SMαA together with either hnRNPA1 or IQGAP1. In brief, paraffin sections of human arteries were deparaffined with xylene and rehydrated with ethanol, and then incubated with 10mM sodium citrate at 100˚C for 10 minutes to retrieve antigens, followed by incubation with an avidin and biotin blocking solution (Avidin Biotin Blocking systems, VectorLab), then a peroxidase blocking solution (3% H2O2), and subsequently 10% normal matched serum (Dako). Thereafter, the sections were incubated with a primary antibody, which was either a rabbit anti-hnRNPA1 (Abcam, ab4791), or a rabbit anti-IQGAP1 antibody (Abcam, ab86064) at 4˚C overnight. The sections were then incubated with a biotin-conjugated goat anti-rabbit secondary antibody (Dako, E0432), followed by an incubation with avidin-conjugated horseradish peroxidase and then with 3,3’-diaminodbenzidine (DAB). The sections were then incubated with a mouse anti-human SMA antibody conjugated with alkaline phosphatase (Sigma, A5691) and then with Fast Red (Sigma). After extensive wash, the sections were counter-stained with hematoxylin solution. After mounting, the sections were examined using an All-in-One Fluorescence Microscope (BZ-X700, Keyence) and images taken using attached camera. Images were processed with Photoshop software (Adobe).

Statistical analysis.

Results are presented as mean ± standard error of the mean (SEM). Statistical analysis was performed using Graphpad Prism5. Shapiro-Wilk Normality Test was used for checking the normality of the data. Two tailed unpaired student's t-test was used for comparisons between 2 groups, or one-way analysis of variance with a post hoc test of LSD was applied when more than two groups were compared if the data display a normal distribution. Pearson's correlation coefficient analyses were conducted to analyse the correlations between the gene expression levels of hnRNAPA1, miR-124 and IQGAP1 in human arterial specimens. $P<0.05$ was considered statistically significant.

Reference:

1. Xiao Q, Zhang F, Grassia G, Hu Y, Zhang Z, Xing Q, Yin X, Maddaluno M, Drung B, Schmidt B, Maffia P, Ialenti A, Mayr M, Xu Q, Ye S. Matrix metalloproteinase-8 promotes vascular smooth muscle cell proliferation and neointima formation. *Arteriosclerosis, thrombosis, and vascular biology*. 2014;34:90-98
2. Wang L, Zheng J, Du Y, Huang Y, Li J, Liu B, Liu CJ, Zhu Y, Gao Y, Xu Q, Kong W, Wang X. Cartilage oligomeric matrix protein maintains the contractile phenotype of vascular smooth muscle cells by interacting with alpha(7)beta(1) integrin. *Circ Res*. 2010;106:514-525
3. Torella D, Iaconetti C, Catalucci D, Ellison GM, Leone A, Waring CD, Bochicchio A, Vicinanza C, Aquila I, Curcio A, Condorelli G, Indolfi C. Microrna-133 controls vascular smooth muscle cell phenotypic switch in vitro and vascular remodeling in vivo. *Circ Res*. 2011;109:880-893
4. Salmon M, Gomez D, Greene E, Shankman L, Owens GK. Cooperative binding of klf4, pelk-1, and hdac2 to a g/c repressor element in the sm22alpha promoter mediates transcriptional silencing during smc phenotypic switching in vivo. *Circ Res*. 2012;111:685-696
5. Chahine MN, Blackwood DP, Dibrov E, Richard MN, Pierce GN. Oxidized ldl affects smooth muscle cell growth through mapk-mediated actions on nuclear protein import. *Journal of molecular and cellular cardiology*. 2009;46:431-441
6. Auge N, Garcia V, Maupas-Schwalm F, Levade T, Salvayre R, Negre-Salvayre A. Oxidized ldl-induced smooth muscle cell proliferation involves the egf receptor/pi-3 kinase/akt and the sphingolipid signaling pathways. *Arteriosclerosis, thrombosis, and vascular biology*. 2002;22:1990-1995
7. Chen Q, Yang F, Guo M, Wen G, Zhang C, Luong le A, Zhu J, Xiao Q, Zhang L. Mirna-34a reduces neointima formation through inhibiting smooth muscle cell proliferation and migration. *J Mol Cell Cardiol*. 2015;89:75-86
8. Huang Y, Lin L, Yu X, Wen G, Pu X, Zhao H, Fang C, Zhu J, Ye S, Zhang L, Xiao Q. Functional involvements of heterogeneous nuclear ribonucleoprotein a1 in smooth muscle differentiation from stem cells in vitro and in vivo. *Stem Cells*. 2013;31:906-917
9. Zhao H, Wen G, Huang Y, Yu X, Chen Q, Afzal TA, Luong le A, Zhu J, Ye S, Zhang L, Xiao Q. Microrna-22 regulates smooth muscle cell differentiation from stem cells by targeting methyl cpg-binding protein 2. *Arterioscler Thromb Vasc Biol*. 2015;35:918-929
10. Yu X, Zhang L, Wen G, Zhao H, Luong LA, Chen Q, Huang Y, Zhu J, Ye S, Xu Q, Wang W, Xiao Q. Upregulated sirtuin 1 by mirna-34a is required for

smooth muscle cell differentiation from pluripotent stem cells. *Cell Death Differ*. 2015;22:1170-1180
11. Luo Z, Wen G, Wang G, Pu X, Ye S, Xu Q, Wang W, Xiao Q. Microrna-200c and -150 play an important role in endothelial cell differentiation and vasculogenesis by targeting transcription repressor zeb1. *Stem Cells*. 2013;31:1749-1762
12. Xiao Q, Zhang F, Lin L, Fang C, Wen G, Tsai TN, Pu X, Sims D, Zhang Z, Yin X, Thomaszewski B, Schmidt B, Mayr M, Suzuki K, Xu Q, Ye S. Functional role of matrix metalloproteinase-8 in stem/progenitor cell migration and their recruitment into atherosclerotic lesions. *Circulation research*. 2013;112:35-47
13. Fang C, Wen G, Zhang L, Lin L, Moore A, Wu S, Ye S, Xiao Q. An important role of matrix metalloproteinase-8 in angiogenesis in vitro and in vivo. *Cardiovascular research*. 2013;99:146-155
14. Liang CC, Park AY, Guan JL. In vitro scratch assay: A convenient and inexpensive method for analysis of cell migration in vitro. *Nat Protoc*. 2007;2:329-333
15. Lindner V, Fingerle J, Reidy MA. Mouse model of arterial injury. *Circ Res*. 1993;73:792-796
16. Xiao Q, Zeng L, Zhang Z, Margariti A, Ali ZA, Channon KM, Xu Q, Hu Y. Sca-1+ progenitors derived from embryonic stem cells differentiate into endothelial cells capable of vascular repair after arterial injury. *Arteriosclerosis, thrombosis, and vascular biology*. 2006;26:2244-2251
17. Zeng L, Xiao Q, Margariti A, Zhang Z, Zampetaki A, Patel S, Capogrossi MC, Hu Y, Xu Q. Hdac3 is crucial in shear- and vegf-induced stem cell differentiation toward endothelial cells. *The Journal of cell biology*. 2006;174:1059-1069
18. Lucerna M, Zernecke A, de Nooijer R, de Jager SC, Bot I, van der Lans C, Kholova I, Liehn EA, van Berkel TJ, Yla-Herttuala S, Weber C, Biessen EA. Vascular endothelial growth factor-a induces plaque expansion in apoe knock-out mice by promoting de novo leukocyte recruitment. *Blood*. 2007;109:122-129
19. de Nooijer R, Verkleij CJ, von der Thusen JH, Jukema JW, van der Wall EE, van Berkel TJ, Baker AH, Biessen EA. Lesional overexpression of matrix metalloproteinase-9 promotes intraplaque hemorrhage in advanced lesions but not at earlier stages of atherogenesis. *Arterioscler Thromb Vasc Biol*. 2006;26:340-346
20. Zhang H, Zhang J, Shen D, Zhang L, He F, Dang Y, Li L. Lentiviral-mediated rna interference of lipoprotein-associated phospholipase a2 ameliorates inflammation and atherosclerosis in apolipoprotein e-deficient mice. *Int J Mol Med*. 2013;31:651-659
21. Zadelaar AS, von der Thusen JH, Boesten LS, Hoeben RC, Kockx MM, Versnel MA, van Berkel TJ, Havekes LM, Biessen EA, van Vlijmen BJ. Increased vulnerability of pre-existing atherosclerosis in apoe-deficient mice following adenovirus-mediated fas ligand gene transfer. *Atherosclerosis*. 2005;183:244-250
22. von der Thusen JH, Fekkes ML, Passier R, van Zonneveld AJ, Mainfroid V, van Berkel TJ, Biessen EA. Adenoviral transfer of endothelial nitric oxide synthase attenuates lesion formation in a novel murine model of postangioplasty restenosis. *Arterioscler Thromb Vasc Biol*. 2004;24:357-362

23. von der Thusen JH, van Vlijmen BJ, Hoeben RC, Kockx MM, Havekes LM, van Berkel TJ, Biessen EA. Induction of atherosclerotic plaque rupture in apolipoprotein e-/- mice after adenovirus-mediated transfer of p53. *Circulation*. 2002;105:2064-2070
24. de Nooijer R, von der Thusen JH, Verkleij CJ, Kuiper J, Jukema JW, van der Wall EE, van Berkel JC, Biessen EA. Overexpression of il-18 decreases intimal collagen content and promotes a vulnerable plaque phenotype in apolipoprotein-e-deficient mice. *Arterioscler Thromb Vasc Biol*. 2004;24:2313-2319
