# Supplementary figures and images for: Novel Pathological Role of hnRNPA1 (Heterogeneous Nuclear Ribonucleoprotein A1) in Vascular Smooth Muscle Cell Function and Neointima Hyperplasia
Source: Arterioscler Thromb Vasc Biol. 2017 Sep 14;37(11):2182–94. doi: 10.1161/ATVBAHA.117.310020 (PMC5660626; doi:10.1161/ATVBAHA.117.310020)

# Graphic Abstract

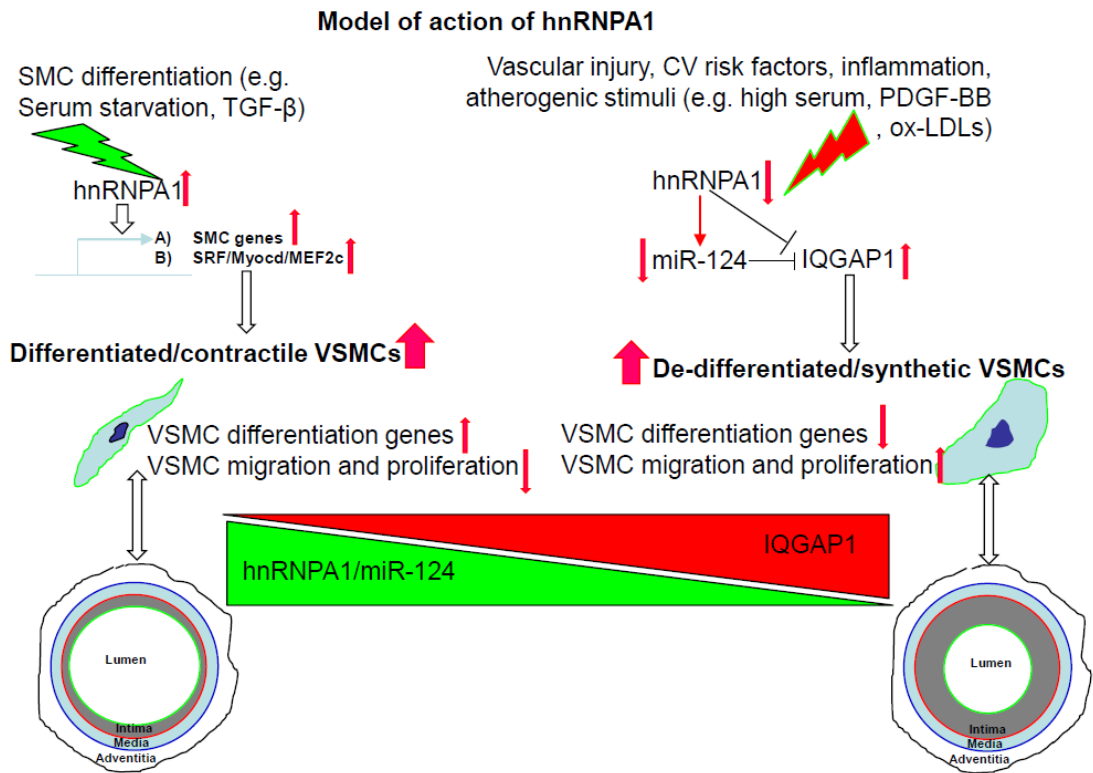

Supplement: Supplementary file 2 [file atv-37-2182-s002.pdf]
