# Supplementary material for: Novel Pathological Role of hnRNPA1 (Heterogeneous Nuclear Ribonucleoprotein A1) in Vascular Smooth Muscle Cell Function and Neointima Hyperplasia
Source: Arterioscler Thromb Vasc Biol. 2017 Sep 14;37(11):2182–94. doi: 10.1161/ATVBAHA.117.310020 (PMC5660626; doi:10.1161/ATVBAHA.117.310020)
Supplement: Supplementary file 3 [file atv-37-2182-s003.pdf]

Supplementary Figures, Figure Legend, and Table

Title: A Novel Pathological role of hnRNPA1 in Vascular Smooth Muscle Cell Functions And Neointima Hyperplasia

Li Zhang, et al.

Supplementary Table I: Primer sets used in the present study

Gene names	Forward (5'-3')	Reverse (5'-3')	Application
U6 snoRNA (mu/hu)	gatgacacgcaaattcgtg	miRNA universal reverse primer (Invitrogen, A11193-051)	RT-qPCR
18s (mu/hu)	aaacggctaccacatccaag	cctccaatggatcctcgtta	RT-qPCR/RIP
Mus/hu miR-24 (mature)	GCTCAGTTCAGCAGGAAC AGA	miRNA universal reverse primer (Invitrogen, A11193-051)	RT-qPCR
Mus/hu miR-124 (mature)	GCGGTGAATGCCAAAAA	miRNA universal reverse primer (Invitrogen, A11193-051)	RT-qPCR
Mus miR-124 (precursor)	CTCTGCGTGTTCACAGCGG AC	CTCTTGGCATTCACCGCGTG	RT-qPCR
Mus miR-124 (5'-primary)	TATTTTCACCGCACTCACG C	CTGCAGTCTTCTGACGGGTG	RT-qPCR/RIP
Mus miR-124 (3'-primary)	ACAAATCCGGCGAACAAT GC	ACCCTTCTCGTGACGTCCT	RT-qPCR/RIP
Mus SMαA	TCCTGACGCTGAAGTATCC GAT	GGCCACACGAAGCTCGTTAT AG	RT-qPCR
Mus h1-Calponin	GGT CCT GCC TAC GGC TTG TC	TCG CAA AGA ATG ATC CCG TC	RT-qPCR
Mus SM-myh11	AAG CAG CCA GCA TCA AGG AG	AGC TCT GCC ATG TCC TCC AC	RT-qPCR
Mus SRF	CCTACCAGGTGTCGGAAT CTGA	TCTGGATTGTGGAGGTGGTA CC	RT-qPCR
Mus Myocardin	TCAATGAGAAGATCGCTC TCCG	GTCATCCTCAAAGGCGAATG C	RT-qPCR
Mus MEF2c	AAGCCAAATCTCCTCCCCC TAT	TGATTCACTGATGGCATCGT GT	RT-qPCR
Mus hnRNPA1	TTCATCCAGTCAGAGAGG TCGC	TGAAGTTCCCTCCTCGACCA A	RT-qPCR
hu hnRNPA1	TGGACCCATGAAGGGAGG AA	GCAAAGTATTGGCCTCCACC	RT-qPCR
Mus IQGAP1	GGAGTTACTGCTGCTACG GT	CTCATTATCCAGGACGGAGC C	RT-qPCR/RIP
Hu IQGAP1	AGAAGTGGCCCAGCATTA CC	TTGGACTGCCAGATTCCACC	RT-qPCR
Mus PCNA	TTGCACGTATATGCCGAG ACCT	ATTGCCAAGCTCTCCACTTG C	RT-qPCR
pmiR-Luc-IQGAP1-WT	GACGAC GAGCTC GCCTCAGTTCACACTCCCT C	CTCCTC ACGCGT GTAAGCACGGCACAACTTCG	IQGAP1 3'UTR reporter clone
pmiR-Luc-IQGAP1-BS^{mut}	ctaaactgtattccaaga ctccttg accaaagtat	atactttggt caaggag tcttggaatacagtttag	miR-124 binding site mutation
pmiR-Luc-IQGAP1-BS^{mut}	cacagcaggcc TGCGTCT gactgagtgggc	gcccactcagtc AGACGCA ggcctgctgtg	ARE/hnRNPA1 binding site mutation
pCDH-hnRNPA1	ATCG GAATTC ATGTCTAAGTCCGAGTCTC CCAAGGAG	ATCG GCGGCCGC TTAGAACCTCCTGCCACTGC CATAG	hnRNPA1 lentribiral

pCDH-GFP	ATCG GAATTC ATGGTGAGCAAGGGCGAG	ATCG GGATCC CTACTTGTACAGCTCGTCCATGC	GFP lentriviral

Supplementary Figures:

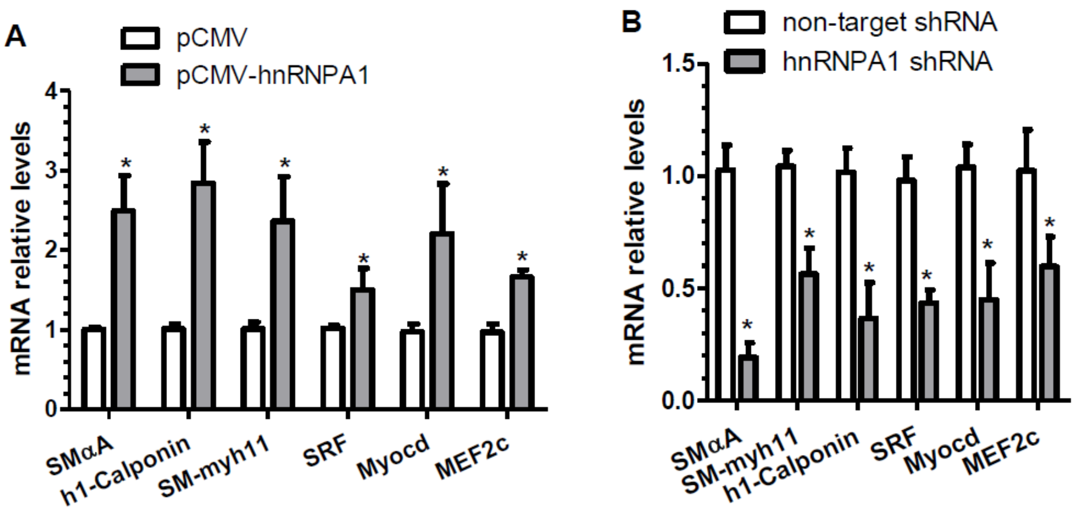

Figure I. hnRNPA1 mediates VSMC contractile gene expression. (A and B) SMC gene expression was up-regulated and down-regulated by hnRNPA1 over-expression (A) and knockdown (B), respectively. The data presented here are mean±S.E.M. of three independent experiments (n=3). *P<0.05 (versus pCMV or non-target shRNA).

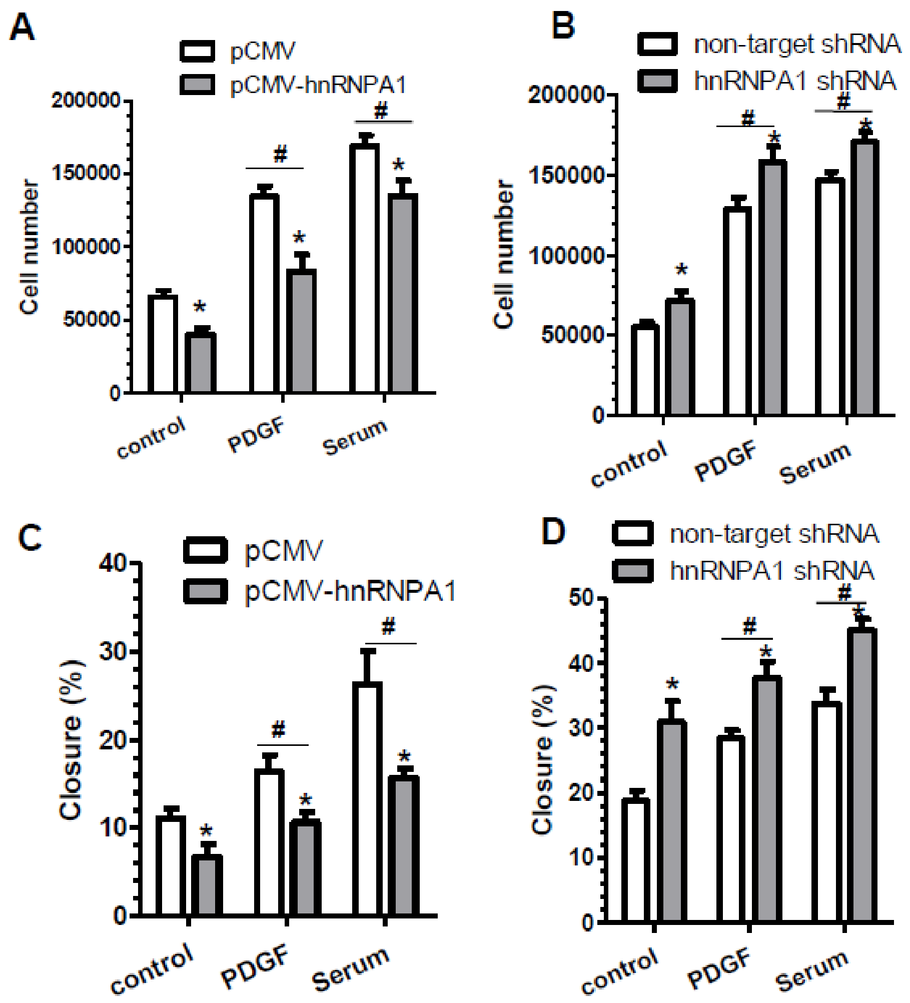

Figure II. hnRNPA1 plays an important role in VSMC proliferation and migration. (A and B) VSMC growth was inhibited and increased by hnRNPA1 over-expression (A) and knockdown (B), respectively. (C and D) Wound-healing analysis of the effects of hnRNPA1 over-expression (C) or knockdown (D) on VSMC migration. For wound-healing assays, the percentage of cell closure or migrated area (%) was calculated as described in the method section and presented here. The data presented here are mean±S.E.M. of three to five independent experiments (n=3~5). *P<0.05 (versus pCMV or non-target shRNA), $^{\#}$P<0.05 (stimuli versus control).

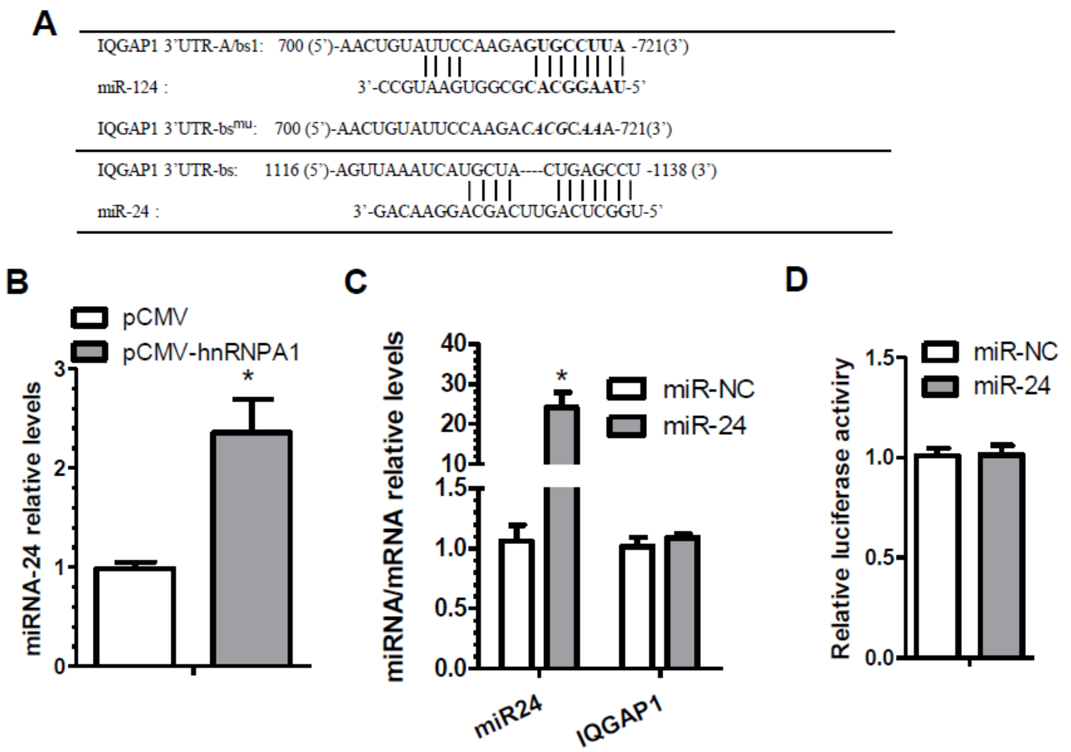

Figure III. IQGAP1 is not a target gene of miR-24 (**A**) The potential wild type binding sites of miR-124 and miR-24 within IQGAP1 3'UTR and the mutant of miR-124 binding site are depicted in this illustration. (B) miR-24 expression level was significantly up-regulated by hnRNPA1. (C-D) IQGAP1 mRNA levels and IQGAP1 3'UTR reporter activity were not regulated by miR-24. The data presented here are mean±S.E.M. of three to four independent experiments (n=3-4). *P<0.05 (versus pCMV).

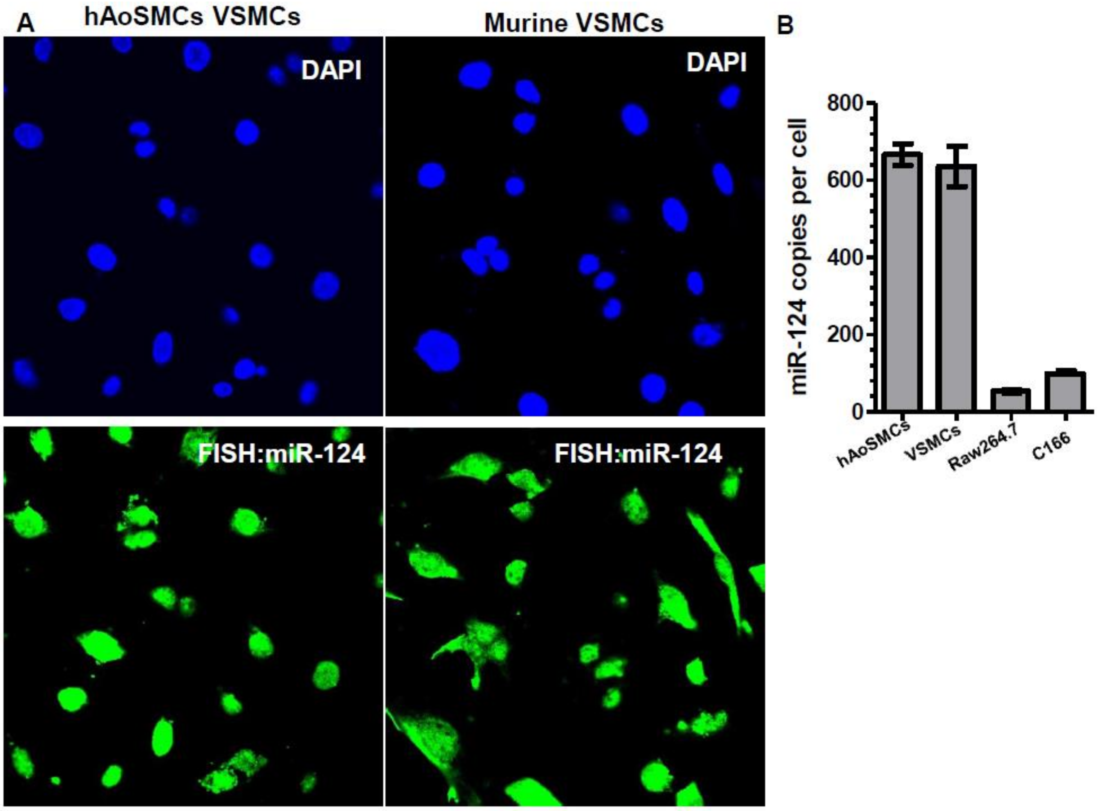

Figure IV. miR-124 expression in VSMCs, macrophages and C166. (**A**) FISH detection of miR-124 in murine and human VSMCs. (B) miR-124 copy numbers (per cell) of miR-124 in VSMCs, macrophages (Raw264.7), and endothelial cells (C166) were quantified by qRT-PCR analyses. The data presented here are representative images or mean±S.E.M. of three independent experiments (n=3-4).

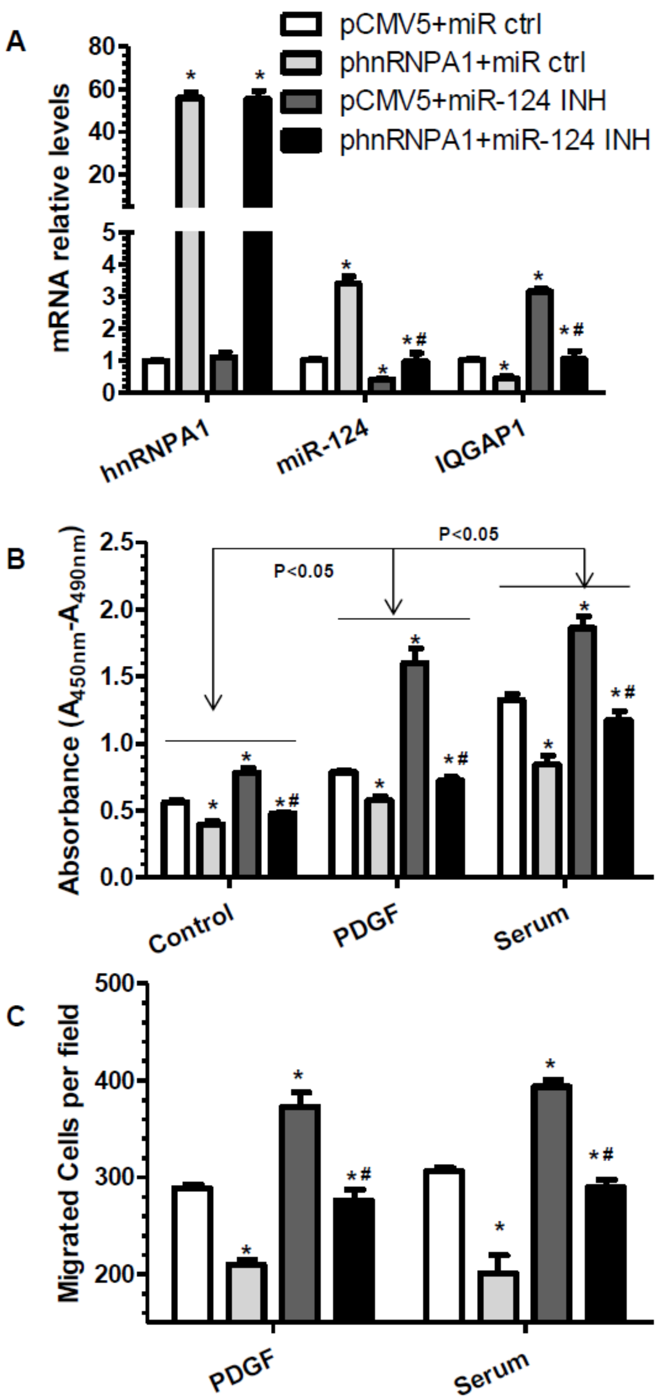

Figure V. miR-124 inhibition abolishes the inhibitory effects of hnRNPA1 over-expression on VSMC proliferation and migration. (A) qRT-PCR analysis of hnRNPA1, miR-124 and hnRNPA1 gene expression. (B) BrdU incorporation assays for VSMC proliferation. (C) Trans-well migration assays. VSMCs were transfected with pCMV5-hnRNPA1 (phnRNPA1), a miR-124 inhibitor (miR-124 INH), or the respective controls (pCMV or miR ctrl) as indicated. Transfected cells were serum-starved for 24 hours and subjected to RT-qPCR analyses (A), proliferation (**B**, BrdU incorporation assay) and migration assays (**C**, trans-well cell migration assay), respectively. The data presented here are mean±S.E.M. of three independent experiments (n=3). *P<0.05 (versus double negative control); #P<0.05 (miR-124 INH versus miR ctrl in the presence of phnRNPA1, 4th versus 2nd column).

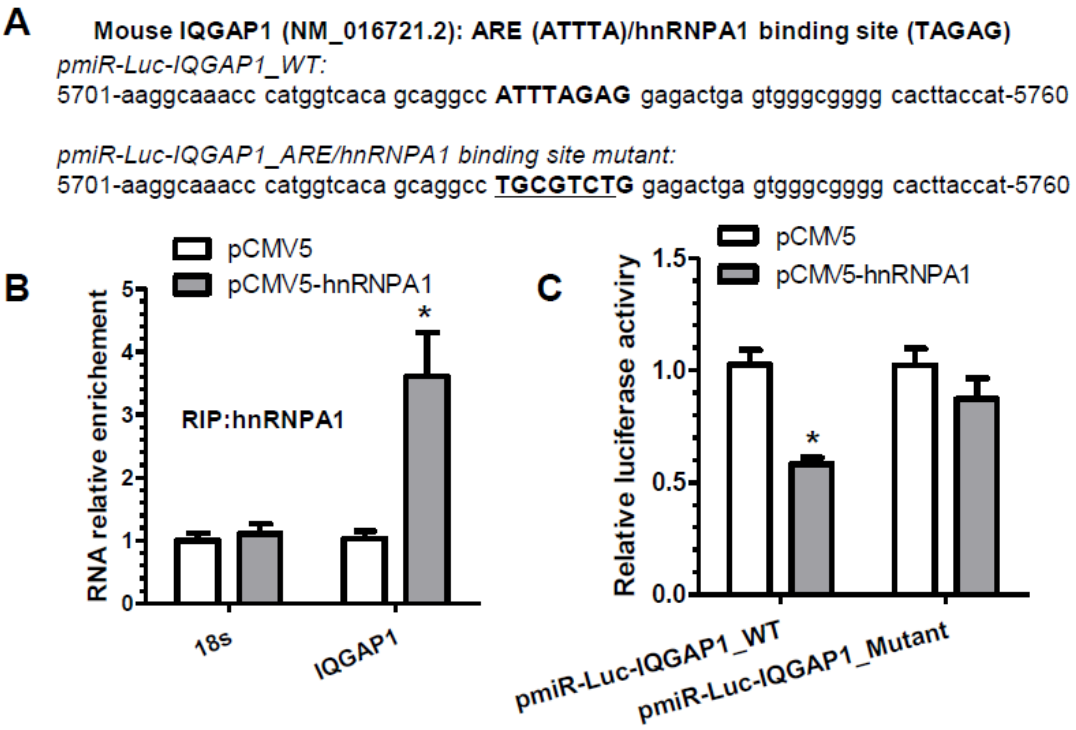

Figure VI. hnRNPA1/ARE binding site is required for hnRNPA1 mediated IQGAP1 mRNA degradation (**A**) The potential wild type binding site of hnRNPA1/ARE within IQGAP1 3'UTR and its mutant are depicted in this illustration. (**B**) RIP assays showed a direct binding between hnRNPA1 and IQGAP1 mRNA. (**C**) hnRNPA1/ARE binding site is required for the inhibitory effects of hnRNPA1 on IQGAP1 3'UTR reporter activity. pCMV-hnRNPA1 or negative control were co-transfected into VSMCs with wild type IQGAP1 3'UTR reporter (**WT**) or the hnRNPA1/ARE binding site mutant (**Mutant**), respectively. Luciferase activity assay were measured at 48 hours post-transfection. The data presented here are mean±S.E.M. of three independent experiments (n=3). *P<0.05 (versus pCMV).

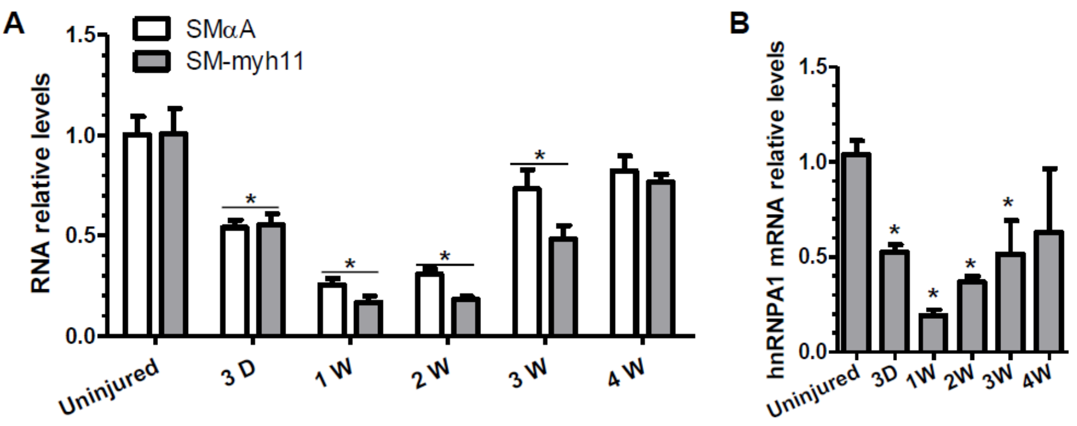

Figure VII. hnRNPA1 expression is decreased in the injured vessels. (A) qRT-PCR analysis for SMαA and SM-myh11 gene expression in carotid arteries isolated from uninjured (normal) or injured mice at the indicated times. (B) hnRNPA1 gene expression is down-regulated during injury-induced arterial remodelling. *P<0.05 (versus control). The data presented here are mean±S.E.M. of three independent experiments (3~5 femoral arteries were pooled for each experiment).

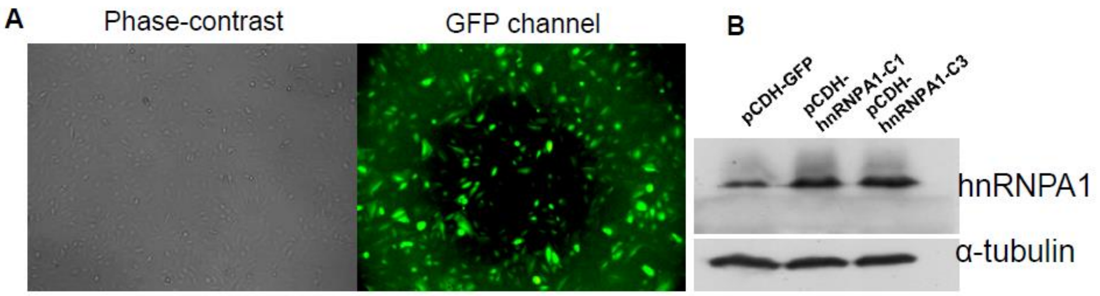

Figure VIII. Infection efficiency of pCDH-GFP or pCDH-hnRNPA1 in mouse VSMCs. (A) Good transduction efficiency was achieved with Lenti-GFP infection in primary VSMCs. (B) Western blot analysis of hnRNPA1 protein expression in primary VSMCs infected with control (pCDH-GFP) or hnRNPA1 over-expression (pCDH-hnRNPA1) lentiviruses. VSMCs infected with pCDH-GFP or pCDH-hnRNPA1 virus were inspected using fluorescence microscopy (A), or harvested and subjected to Western Blot (B) analyses, respectively, to detect the expression of hnRNPA1 in VSMCs.

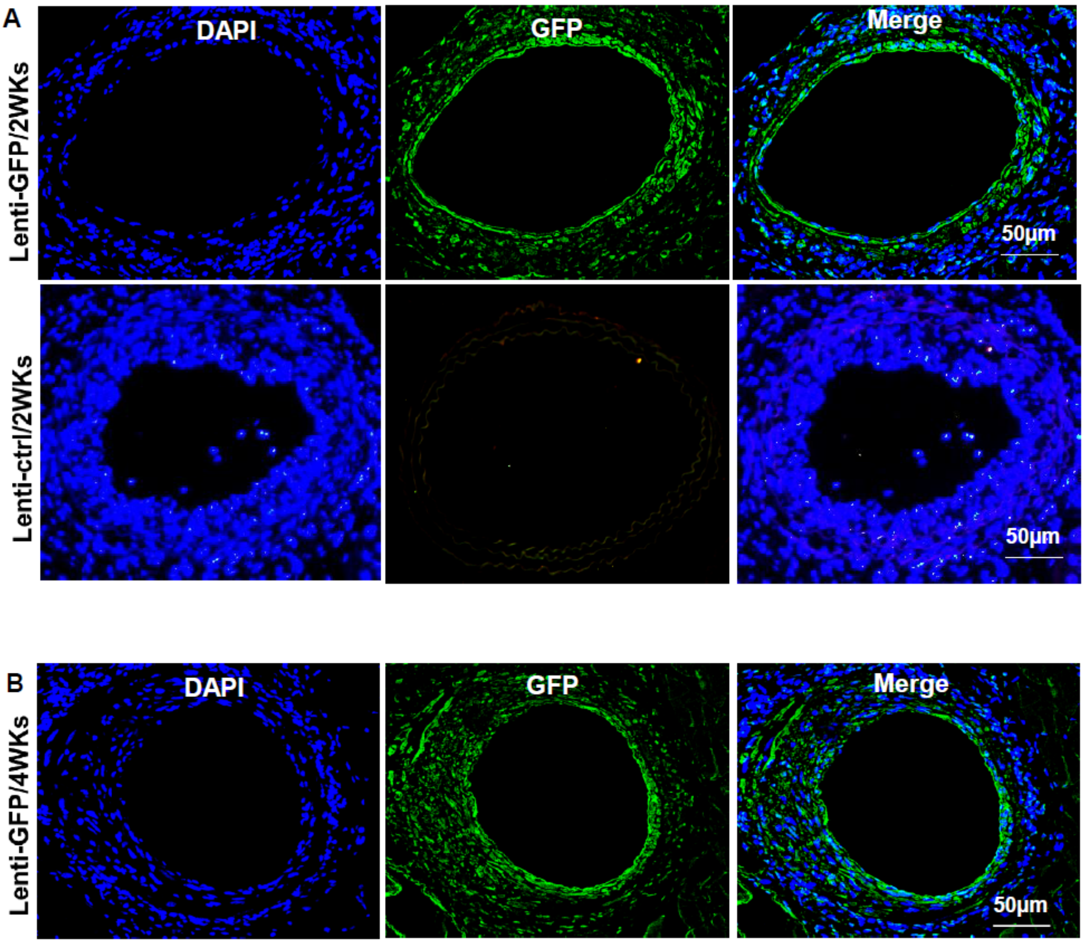

Figure IX. Local gene delivery into VSMCs within injured arterial wall by lentivirus. (A) Representative images showing GFP expression in the injured carotid arteries infected with GFP expression (Lenti-GFP) or control Lentivirus (Lenti-ctrl) at two weeks post-treatment. (B) **Representative images** showing GFP expression in the injured carotid arteries infected with Lenti-GFP at four weeks post-treatment.

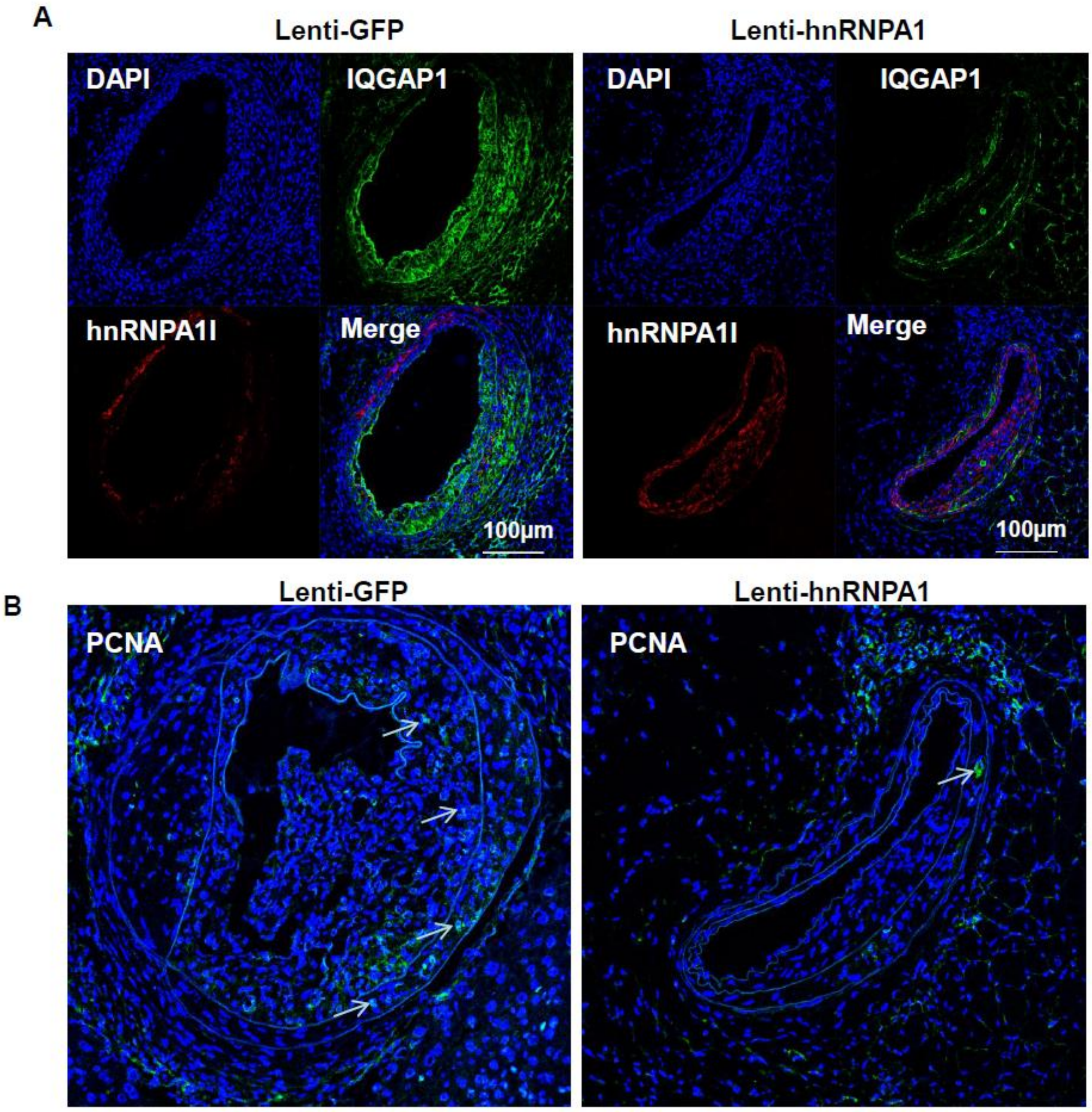

Figure X. Locally enforced expression of hnRNPA1 inhibits IQGAP1 expression and reduces PCNA-positive cells in the injured vessels. (A) Representative images showing hnRNPA1 and IQGAP1 expression in the injured carotid arteries infected with GFP expression (Lenti-GFP) or control Lentivirus (Lenti-ctrl) at two weeks post-treatment. (B) Representative images showing VSMC proliferation (PCNA-positive cells) in the injured carotid arteries infected with GFP expression (Lenti-GFP) or control Lentivirus (Lenti-ctrl) at two weeks post-treatment. Paraffin sections from both groups (n=6 mice) were prepared and stained with hnRNPA1/IQGAP1 (A) or PCNA (B) antibody, respectively. Representative images were presented here. Notes: white arrows indicate PCNA-positive cells.

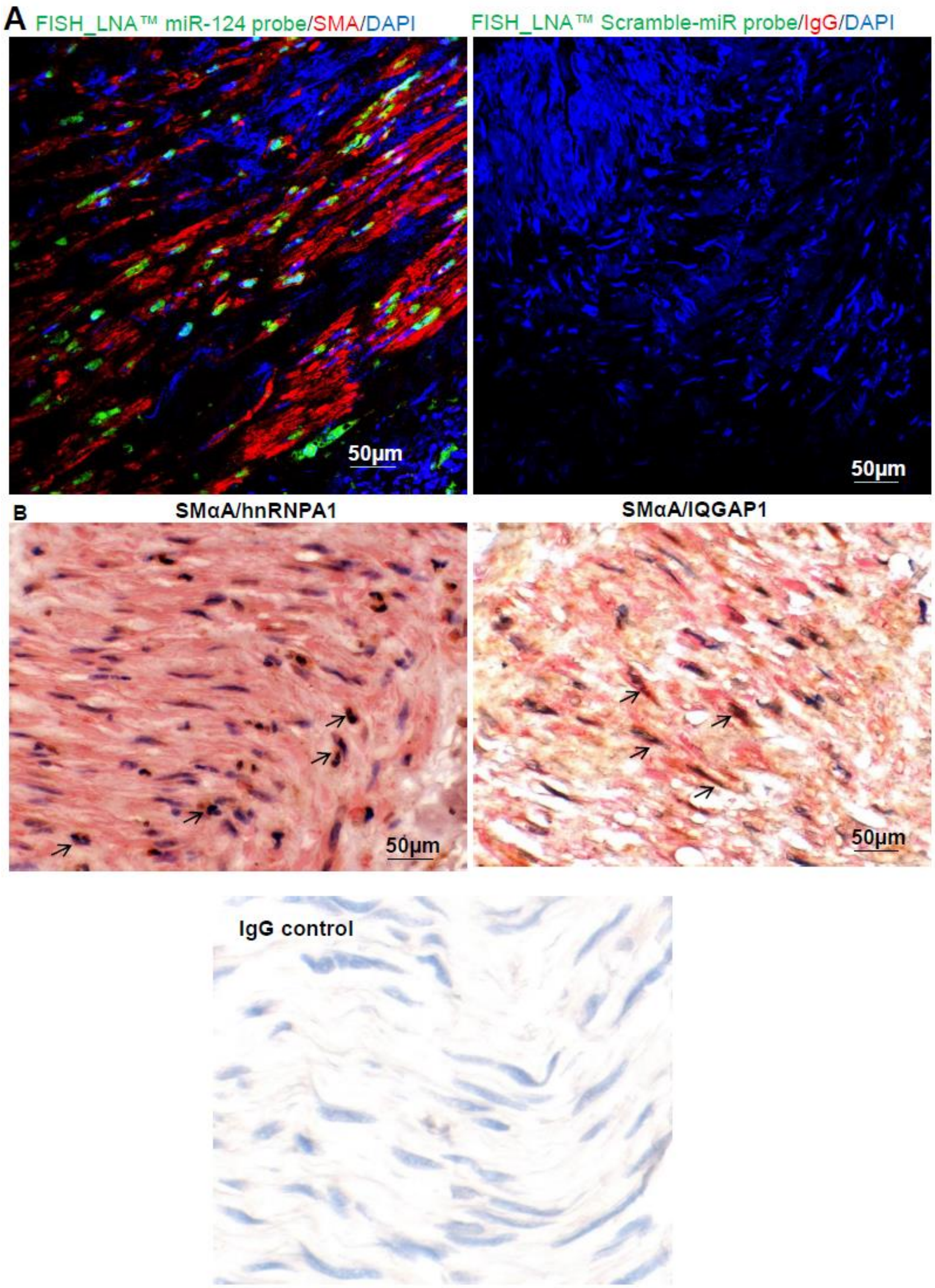

Figure XI. Detection of miR-124, hnRNPA1 and IQGAP1 in VSMCs within human diseased vessels. (A) Human femoral arterial specimens were collected, and subjected to FISH assays to detect miR-124 expression (green), followed by immunofluorescence staining with smooth muscle α-actin (SmαA) antibody (Red). (B) Double immunohistochemistry analyses with antibodies against SMαA and hnRNPA1 or IQGAP1 were conducted with sections from human femoral arterial specimens. Red color (AKP/fast red) indicates SMαA staining, and dark brown color with DAB indicates hnRNPA1 or IQGAP1 staining. Representative images were presented here. Notes: black arrows indicate double positive cells.

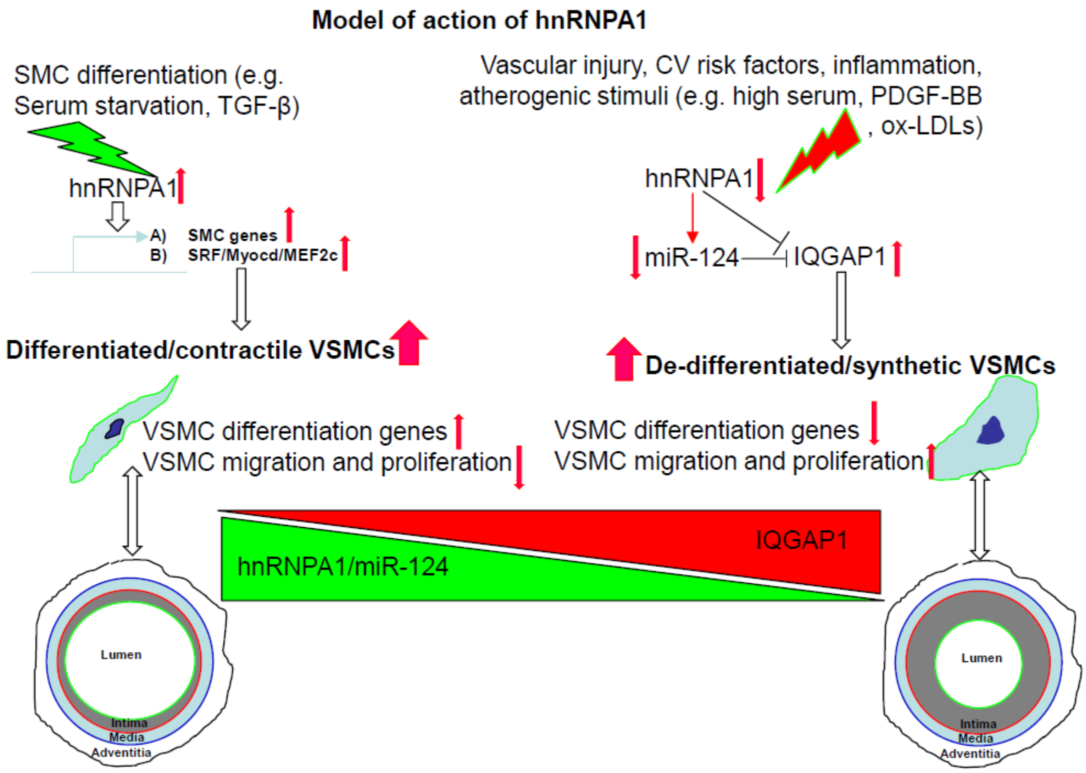

Figure XII. Model of action of hnRNPA1 in modulating VSMC functions and neointima formation In response to VSMC differentiation stimuli (e.g. serum starvation and TGF-β) or within normal healthy arteries, hnRNPA1 expression is increased in VSMCs, resulting in SMC differentiation gene programme activation through two transcriptional mechanisms: transcriptional up-regulation of SMC contractile genes (SMαA, SM22α, Calponin and SM-MHC) and SMC transcription factors (SRF, Myocardin/Myocd and MEF2C), as reported in our previous study[1]. Consequently, VSMCs within blood vessel wall express high level of hnRNPA1 and maintain its contractile phenotype. On the other hand, in response to atherogenic stimuli [e.g. vascular injury, cardiovascular (CV) risk factors, inflammation, high serum, PDGF-BB, oxidized low-density lipoprotein (oxLDL)], hnRNPA1 expression in VSMCs or injured arteries is inhibited. Decreased hnRNPA1 up-regulates IQGAP1 through two mechanisms (modulating miR-124 biogenesis, and miR-124 and hnRNPA1/ARE binding site within 3'UTR of IQGAP1). Increased IQGAP1 promotes VSMC proliferation and migration, resulting in neointima formation.
